# Dynamics of the gut microbiome, IgA response, and plasma metabolome in the development of pediatric celiac disease

**DOI:** 10.1186/s40168-022-01429-2

**Published:** 2023-01-13

**Authors:** Khyati Girdhar, Yusuf Dogus Dogru, Qian Huang, Yi Yang, Vladimir Tolstikov, Amol Raisingani, Martina Chrudinova, Jaewon Oh, Kristina Kelley, Jonas F. Ludvigsson, Michael A. Kiebish, Noah W. Palm, Johnny Ludvigsson, Emrah Altindis

**Affiliations:** 1 Boston College Biology Department, Chestnut Hill, MA 02467 USA; 2 Department of Immunobiology, Yale University School of Medicine, New Haven, CT 06510 USA; 3 BERG, LLC, Framingham, MA USA; 4 Department of Medical Epidemiology and Biostatistics, Karolinska Institutet, Stockholm, Sweden; 5 Department of Paediatrics, Örebro University Hospital, Örebro, Sweden; 6 Crown Princess Victoria Children’s Hospital, Division of Pediatrics, Department of Biomedical and Clinical Sciences, Linköping University, 58185 Linköping, SE Sweden

**Keywords:** Celiac disease, Gut microbiota, IgA sequencing, Metabolites, Cytokines, Taurodeoxycholic acid, Qa-1, NKG2D

## Abstract

**Background:**

Celiac disease (CD) is an autoimmune disorder triggered by gluten consumption. Almost all CD patients possess human leukocyte antigen (HLA) DQ2/DQ8 haplotypes; however, only a small subset of individuals carrying these alleles develop CD, indicating the role of environmental factors in CD pathogenesis. The main objective of this study was to determine the contributory role of gut microbiota and microbial metabolites in CD onset. To this end, we obtained fecal samples from a prospective cohort study (ABIS) at ages 2.5 and 5 years. Samples were collected from children who developed CD after the final sample collection (CD progressors) and healthy children matched by age, HLA genotype, breastfeeding duration, and gluten-exposure time (*n*=15–16). We first used 16S sequencing and immunoglobulin-A sequencing (IgA-seq) using fecal samples obtained from the same children (i) 16 controls and 15 CD progressors at age 2.5 and (ii) 13 controls and 9 CD progressors at age 5. We completed the cytokine profiling, and plasma metabolomics using plasma samples obtained at age 5 (*n*=7–9). We also determined the effects of one microbiota-derived metabolite, taurodeoxycholic acid (TDCA), on the small intestines and immune cell composition in vivo.

**Results:**

CD progressors have a distinct gut microbiota composition, an increased IgA response, and unique IgA targets compared to healthy subjects. Notably, 26 plasma metabolites, five cytokines, and one chemokine were significantly altered in CD progressors at age 5. Among 26 metabolites, we identified a 2-fold increase in TDCA. TDCA treatment alone caused villous atrophy, increased CD4+ T cells, Natural Killer cells, and two important immunoregulatory proteins, Qa-1 and NKG2D expression on T cells while decreasing T-regulatory cells in intraepithelial lymphocytes (IELs) in C57BL/6J mice.

**Conclusions:**

Pediatric CD progressors have a distinct gut microbiota composition, plasma metabolome, and cytokine profile before diagnosis. Furthermore, CD progressors have more IgA-coated bacteria and unique targets of IgA in their gut microbiota. TDCA feeding alone stimulates an inflammatory immune response in the small intestines of C57BJ/6 mice and causes villous atrophy, the hallmark of CD. Thus, a microbiota-derived metabolite, TDCA, enriched in CD progressors’ plasma, has the potential to drive inflammation in the small intestines and enhance CD pathogenesis.

Video Abstract

**Supplementary Information:**

The online version contains supplementary material available at 10.1186/s40168-022-01429-2.

## Introduction

Celiac disease (CD) is a gluten-induced autoimmune disorder that is predicted to affect 1 in 100 individuals worldwide [1]. Almost all CD patients possess HLA-DQ2 or HLA-DQ8. However, while 20–40% of the population in Europe and the USA carries these alleles, only 1% of individuals develop the disease [2]. The incidence and prevalence of CD have continued to increase over the past 15 years [3–5], and genetics alone cannot explain the increase. Various environmental factors are implicated in CD development [6]. Gut microbiome studies have observed altered microbial and fecal metabolite compositions [7–11] in both infant and adult CD patients [8–13]. Studies have yet to identify any causal or contributory link between the gut microbiome and the disease onset.

In this study, we used fecal and plasma samples from a prospective cohort of CD progressors who developed the disease after the last sample collection and healthy children matched by age, HLA genotype, breastfeeding duration, and gluten-exposure duration. To assess the gut microbiota and plasma metabolome alterations in pediatric CD onset, we used fecal samples obtained at ages 2.5 and 5, representing the two important stages of gut microbiota development [14]. This analysis revealed that CD progressors already had significant alterations in their gut microbiome composition, a unique IgA response against gut bacteria, and a distinct plasma metabolome and cytokine profile before diagnosis. In addition, we showed that a microbiota-derived metabolite, TDCA, that is increased two-fold in CD progressors can cause villous atrophy and exacerbate inflammation in the small intestines of C57BJ/6 mice. Thus, TDCA may play a key role in pediatric CD onset.

## Methods

### Human fecal samples

The fecal samples were obtained from subjects in the All Babies in Southeast Sweden (ABIS) cohort. The ABIS study was ethically approved by the Research Ethics Committees of the Faculty of Health Science at Linköping University, Sweden (Ref. 1997/96287 and 2003/03-092) and the Medical Faculty of Lund University, Sweden (Dnr 99227, Dnr 99321). All children born in southeast Sweden between 1 October 1997 and 1 October 1999 (*n*=21,700) were eligible to participate, with 17,055 families electing to partake after informed consent. Among them, 181 children developed CD by Dec 31, 2017. Fresh fecal samples were collected at home or the clinic and frozen immediately. Questionnaires and biological samples were collected at ages 1, 2.5, 5, and 8 years of age. Later, follow-up and web questionnaires were conducted at ages 11–13 years and 17–18 years, respectively. The questionnaire was completed by the parents to collect participants’ health information, including, but not limited to, breastfeeding duration, antibiotic use, and gluten exposure time. In this study, we matched CD progressors with healthy controls who had not developed any autoimmune disease based on age, sex, and HLA type. Using these matching criteria, we were able to select a total of 33 children. Among 16 CD progressors, one child developed CD before age 2.5, and four children developed CD after age 2.5, but before age 5. Therefore, we used a total of 31 (16 controls and 15 CD progressors) fecal samples at age 2.5 and a total of 22 (13 controls and 9 CD progressors) fecal samples at age 5. Of the 16 CD progressors, five of them developed CD before age 5. In addition, we used a total of 16 plasma samples collected at age 5 (9 controls and 7 CD progressors) for plasma metabolomics and cytokine profiling (Additional file 1: Figure S1 and Additional file 2: Table S1). Diagnosis of CD was confirmed at least twice, according to the Swedish National Diagnosis Register [15]. Until 2012, the CD was diagnosed by both serology and small intestinal biopsy. Both records were registered on the Swedish national inpatient register [15].

### 16S rRNA gene sequencing

16S rRNA sequencing of the V4 region was performed on a Miseq platform with barcoded primers. Briefly, all bacterial samples were suspended in 90μl of MicroBead Lysis Solution with 10% RNAse-A and sonicated in a water bath at 50°C for 5 min. Samples were transferred to a plate containing 50μl of Lysing Matrix B (MP Biomedicals) and homogenized by bead-beating for 5 min. After centrifugation (4122 × *g*, 4°C, 6min), the supernatant was transferred to 2-ml deep-well plates (Axygen Scientific). Bacterial DNA from the samples was extracted and purified using a MagAttract Microbial kit (QIAGEN), following instructions provided by the manufacturer. PCR was performed to amplify the V4 region of 16S ribosomal RNA (33 cycles) in duplicate (3μl purified DNA per reaction; Phusion DNA polymerase, New England Bioscience) [16]. After amplification, PCR products were then normalized with a SequalPrep^TM^ normalization plate kit (ThermoFisher Scientific) and pooled. The pooled library concentration was calculated by using an NGS Library Quantification Complete kit (Roche 07960204001) and then loaded on a Miseq sequencer. Illumina Miseq Reagent Kit V2 (500 cycles) was used to generate 2×250bp paired-end reads. The raw reads were demultiplexed in Qiime1 (version 1.9). The sequencing yielded a mean of 30,471 reads per sample.

### IgA+ and IgA− bacteria separation

IgA+ and IgA*−* bacteria separation was performed as previously described [16]. Briefly, frozen human fecal samples were suspended in phosphate-buffered saline (PBS) (100mg/mL) and were homogenized using Fast Prep Lysing Matrix D with ceramic beads (MP Biomedicals). The samples were bead-beaten for 7s (Minibeadbeater; Biospec) and then centrifuged 50×*g* for 10 min at 4°C. Fecal bacteria in the supernatants were collected (200μl/sample) and washed three times with 500μl PBS containing 1% (w/v) bovine serum albumin (BSA, American Bioanalytical) and centrifuged (6000 × rpm, 4°C, 5 min). A sample of this washed bacterial suspension (50μl) was collected as the pre-sorting sample for 16S sequencing analysis. After washing, bacterial pellets were re-suspended in 50μl blocking buffer (PBS containing 1% (w/v) BSA and 20% normal mouse serum (Jackson ImmunoResearch), incubated for 20 min on ice, and stained with 100μl PE-conjugated mouse anti-human IgA (1:40; Miltenyi Biotec clone IS11-8E10) for 30 min on ice. Samples were subsequently washed three times with 500μl BSA containing 1% (w/v) before flow cytometry analysis or cell separation. PE anti-human IgA-stained bacteria were incubated with anti-PE magnetic-activated cell sorting (MACS) beads (Miltenyi Biotec) (1:5) for 30 min on ice and then separated by a custom magnetic plate for 10 min on ice. Fecal bacteria bound to the magnetic plate were collected as IgA+ samples for 16S sequencing analysis. Stained and MACS bead-bound bacteria unbound to magnet plate were collected (20~40μl) and passed through MACS molecular columns (Miltenyi Biotec) (one sample/column), followed by flushing with 480μl PBS containing 1% (w/v) BSA. The total column pass-through (~1 ml) was saved as IgA− samples for 16S sequencing analysis.

### Fecal IgA flow cytometry analysis

Bacterial cells were isolated from fecal samples as described in the IgA+ and IgA− bacteria separation method section of this manuscript. Bacteria were stained with PE anti-human IgA antibodies (1:100; Miltenyi Biotec clone IS11-8E10) for 30 min on ice. After washing twice, bacteria were stained with TO-PRO®-3 (ThermoFisher Scientific) to identify bacteria from fecal debris or particles. Stained bacteria were analyzed by a BD FACSAria^TM^ IIIu cell sorter (Becton-Dickinson) as previously described [17] as TO-PRO®-3^+^IgA^±^ cells.

### Plasma metabolomic analysis

Plasma samples for metabolomics analysis were prepared as previously described [18, 19]. Metabolite extraction from plasma was achieved using a mixture of isopropanol, acetonitrile, and water at a ratio of 3:3:2 v/v. Extracts were divided into three parts: 75 μl for gas chromatography combined with time-of-flight high-resolution mass spectrometry, 150 μl for reverse-phase liquid chromatography coupled with high-resolution mass spectrometry, and 150 μl for hydrophilic interaction chromatography with liquid chromatography and tandem mass spectrometry, and analyzed as previously described [18, 19]. We used the NEXERA XR UPLC system (Shimadzu, Columbia, MD, USA) coupled with the Triple Quad 5500 System (AB Sciex, Framingham, MA, USA) to perform hydrophilic interaction liquid chromatography analysis, the NEXERA XR UPLC system (Shimadzu, Columbia, MD, USA) coupled with the Triple TOF 6500 System (AB Sciex, Framingham, MA, USA) to perform reverse-phase liquid chromatography analysis, and an Agilent 7890B gas chromatograph (Agilent, Palo Alto, CA, USA) interfaced to a time-of-flight Pegasus HT mass spectrometer (Leco, St. Joseph, MI, USA) for gas chromatography. The GC system was fitted with a Gerstel temperature-programmed injector-cooled injection system (model CIS 4). An automated liner exchange (ALEX) (Gerstel, Muhlheim an der Ruhr, Germany) was used to eliminate cross-contamination from the sample matrix that was occurring between sample runs. Quality control was performed using metabolite standards, mixture, and pooled samples. A standard quality control sample containing a mixture of amino and organic acids was injected daily to monitor the mass spectrometer response. A pooled quality control sample was obtained by taking an aliquot of the same volume from all samples of the study and injecting daily with a batch of analyzed samples to determine the optimal dilution of the batch samples and validate metabolite identification and peak integration. Collected raw data were manually inspected, merged, inputted, and normalized by the sample median. Metabolite identification was performed using in-house authentic standards analysis. Metabolite annotation was used utilizing recorded retention time, and retention indexes recorded MS^n^ and HRAMS^n^ data matching with METLIN, NIST MS, Wiley Registry of Mass Spectral Data, HMDB, MassBank of North America, MassBank Europe, Golm Metabolome Database, SCIEX Accurate Mass Metabolite Spectral Library, MzCloud, and IDEOM databases.

### Metabolite pathway analysis

Metabolomic data were analyzed as previously described by Tolstikov et al. [20]. Identified metabolites were subjected to pathway analysis with MetaboAnalyst 4.0, using a Metabolite Set Enrichment Analysis (MSEA) module, which consists of an enrichment analysis relying on measured levels of metabolites and pathway topology and provides visualization of the identified metabolic pathways. Accession numbers of detected metabolites (HMDB, PubChem, and KEGG Identifiers) were generated, manually inspected, and utilized to map the canonical pathways. MSEA was used to interrogate functional relation, which describes the correlation between compound concentration profiles and clinical outcomes.

### Bioinformatic analysis and statistics

Microbial diversity and statistical analyses were performed by filtering and trimming the bacterial 16s rRNA amplicon sequencing reads, and sample inference that turns amplicon sequences into an Amplicon Sequence Variant (ASVs) table was performed by dada2 [21] using the Ribosomal Database Project Training Set 16 [22]. Exploratory and inferential analyses were performed in R (version 4.1.2) using phyloseq [23] and vegan [24], which includes non-metric multidimensional scaling (NMDS) analysis using Bray–Curtis dissimilarity, Principle Components Analysis (PCA), alpha and beta diversity estimates, and taxa agglomeration. Statistical significance was assessed by ANOVA for alpha diversity and PERMANOVA for Bray-Curtis dissimilarity. Differential ASVs abundance was assessed per time point by edgeR [25] with two-sided empirical Bayes quasi-likelihood *F* tests. *P* values were corrected by using the Benjamini-Hochberg false discovery rate (FDR), and FDR < 0.05 was considered a statistically significant [26]. For IgA-sorted samples, an IgA coating index (ICI) was calculated for each taxon. ICI is calculated as the ratio of the relative abundance of taxa in the IgA-positive sample and the relative abundance of the taxa in the IgA-negative sample. Taxa with ICI values lower than 1 were discarded for the heatmap, except where ICI values for a particular taxon were as follows: (ICI_Control_ <1 and ICI_Celiac_ > 1) or (ICI_Control_ >1 and ICI_Celiac_ <1). The results were normalized based on rows (ICI scores) and visualized using the pheatmap package (RRID:SCR_016418).

### Animals

C57BL/6J mice were maintained and bred in the Boston College Animal Care Facility. All the animal experiments were conducted following the regulations and ethics guidelines of the National Institute of Health and were approved by the IACUC of Boston College (Protocol No.#B2019-003 and 2019-004). The mice were maintained under specific pathogen-free conditions in a 12-h dark/light cycle with free access to autoclaved water and bedding. After weaning, 3-week-old littermate-matched mice were divided into two groups: (1) the control group, which was provided with a chow diet, and (2) the TDCA group, which was provided with a chow diet containing 0.4% TDCA (wt/wt). After maintaining mice on these diets for 10 weeks, mice were sacrificed, and primary cells and organs were collected for further analysis.

### Histopathological sectioning and staining

The parafilm sections of the duodenum and ileum were dried, stained with a hematoxylin and eosin (H&E) staining kit (Vector Laboratory), and analyzed using the upright microscope (Zeiss AxioImager Z2). The villi/crypt ratio and plasma cells were quantified using Fiji/ImageJ software.

### Primary cell isolation and flow cytometry

Cells were isolated from the spleen and mesenteric lymph nodes (MLN) via mechanical disruption, and lamina propria (LPs) and Peyer’s patches (PPs) were isolated as described previously [27]. Briefly, the intestines were washed with PBS, cut transversally into small 1-cm pieces, and fixed in formalin. The remaining intestines were cut longitudinally to expose the inner epithelium layer and were washed with phosphate buffer saline (PBS) containing 2% fetal bovine serum (FBS) 2–3 times to get rid of feces. The cut pieces of the intestines were stirred in freshly prepared dithiothreitol (DTE) solution for 20 min two times. After 20 min, IELs were collected from the supernatant, filtered using a 70μm filter, and then passed through a 44/67 Percoll gradient. The intestine pieces were stirred in EDTA solution for 30 min a total of two times, then washed with complete media. To isolate LPs and PPs cells, the cleaned intestines and PPs were resuspended in collagenase solution. Cells were collected from the supernatant by passing through a 70-μm filter and then passed through a 44/67 Percoll gradient. The cell suspension was then washed and surface labeled with the appropriate fluorochrome-conjugated monoclonal antibodies, as mentioned in Additional file 2: Table S6. The results were assessed using flowjo10 software.

## Results

### Celiac disease progressors have a distinct gut microbiota composition

**Fig. 1:**
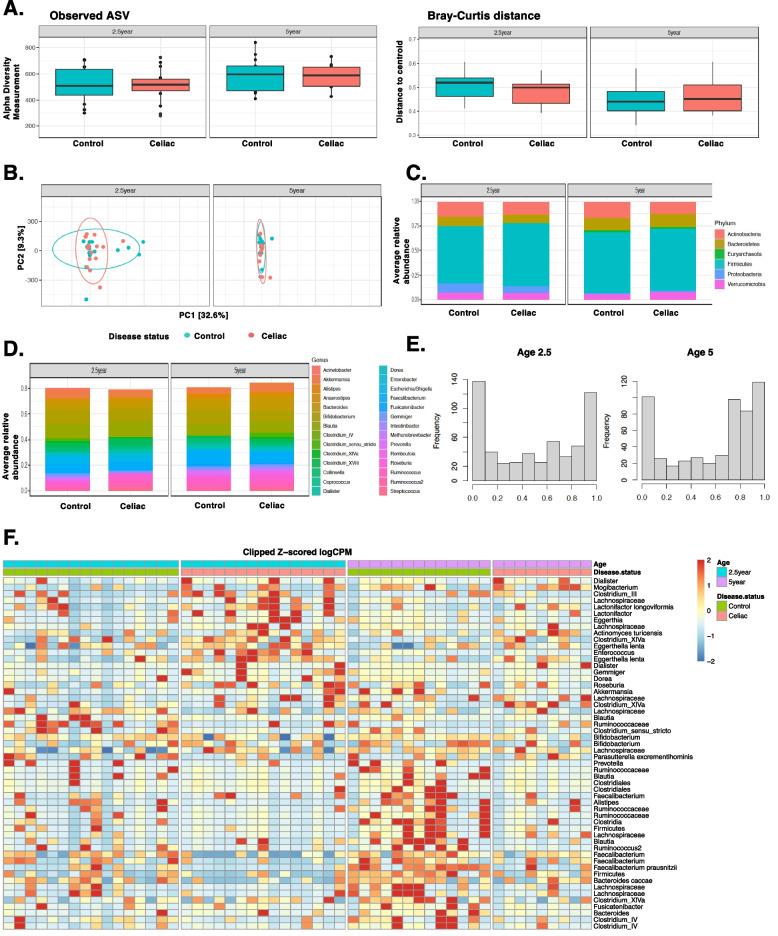
Gut microbiota of children developing CD was significantly altered at the ASV level. **A** Box plots showing the comparison between CD progressors (*n*=9–15) and healthy controls (*n*=13–16): the alpha diversity measured by observed ASVs (left panel) and the beta diversity measured by Bray–Curtis dissimilarity (right panel). Statistical analysis was performed using ANOVA (alpha diversity) and PERMANOVA (Bray-Curtis distance). **B** Principal component analysis (PCA) ordination of sample similarity/dissimilarity between CD progressors and healthy controls at age 2.5 and 5 years old. Each circle represents an individual sample, control (green bar) and celiac (red bar). **C**, **D** Average relative abundance of bacterial phylum (upper panel) or genera (lower panel) of greater than 1% abundance (proportion) between the gut microbiota of CD progressors and healthy controls at age, 2.5, and 5 years old (taxa average relative abundance>1%). Statistical analysis was performed using two-tailed *t* tests with the Benjamini and Hochberg method to control the false discovery rate (FDR). **E** Empirical Bayes quasi-likelihood *F* tests analysis for the comparisons of gut microbiota ASVs between CD progressors and healthy controls at ages 2.5 and 5 years old. Frequency: number of ASVs. **F** Heat map showing the relative abundance of the top ASVs significantly different between CD progressors and healthy controls. Each column represents an individual participant, and each row represents an ASV

We first determined gut microbiome composition differences between CD progressors (*n*=15, age 2.5; *n*=9, age 5) and healthy-matched controls (*n*=16, age 2.5; *n*=13, age 5) using 16S rRNA gene sequencing. The characteristics of the subjects are described in Additional file 1: Figure S1 and Additional file 2: Table S1. In total, we identified 575 amplicon sequence variants (ASVs) (Additional file 2: Table S2). Alpha and beta diversity were comparable between CD progressors and healthy subjects at ages 2.5 and 5 (Fig. 1A). Principal component analysis (PCA) did not show a significant separation (Fig. 1B), and relative abundance analyses did not identify differences among the phylum or genera levels (Fig. 1C and D, Additional file 2: Table S3). However, we identified significant differences at the ASV level in both ages using a stringent statistical threshold to report any significant finding (FDR <0.05, *p* <0.01). Specifically, 117 ASVs were different at age 2.5, and 71 ASVs were different at age 5 (Fig. 1E and Additional file 2: Table S2). The most significantly enriched ASVs, either in CD progressors’ samples or healthy subjects, are shown in Table 1 and Additional file 1: Figure S2. The heat map shows a clear separation between CD progressors and healthy subjects at both ages (Fig. 1F).

**Table 1:** Top ASVs enriched and/or IgA targeted in CD progressors and controls

Age 2.5				Age 5			
Group	ASV	FC	FDR	Group	ASV	FC	FDR
**Presort (Control vs Celiac disease)**							
**Enriched in CD progressors**	*Dialister*	614	0.001	**Enriched in CD progressors**	*Dialister propionicifaciens*	457	0.010
	*Gemmiger*	257	0.001		*Dialister*	1680	0.009
	*Ruminococcaceae*	152	0.001		*Roseburia*	157	0.019
	*Dialister propionicifaciens*	48.5	0.008		*Clostridium sensu stricto*	74.9	0.025
	*Porphyromonas asaccharolytica*	20.9	0.018		*Shigella dysenteriae*	19.4	0.027
**Enriched in Healthy Controls**	*Methanobrevibacter*	-818	0.001	**Enriched in Healthy Controls**	*Akkermansia*	-1290	0.023
					*Prevotella*	-551	0.021
	*Clostridium sensu stricto*	-582	0.001		*Holdemanella*	-387	0.031
	*Bacteroides eggerthii*	-553	0.001		*Catenibacterium mitsuokai*	-197	0.036
	*Bacteroides uniformis*	-157	0.001		*Bacteroides stercoris*	-171	0.026
	*Bacteroides stercoris*	-128	0.003		*Bacteroides massiliensis*	-154	0.046
**Post sort (IgA+ vs IgA-)**							
**IgA Target in CD progressors**	*Elizabethkingia*	134	6.95E-08	**IgA Target in CD progressors**	*Clostridium IV*	4360	0.0002
	*Brucella*	87.4	6.95E-08		*Streptococcus*	99.3	0.027
	*Coprococcus comes*	69.1	0.002		*Brucella*	45.9	0.0002
	*Bifidobacterium bifidum*	32.5	0.016		*Elizabethkingia*	27	0.030
**IgA Target in Healthy Controls**	*Bifidobacterium*	190	0.002	**IgA Target in Healthy Controls**	*Clostridium IV*	838	3.58E-05
	*Bacteroides clarus*	140	0.004		*Gemmiger*	141	0.015
	*Catenibacterium mitsuokai*	66.6	0.014		*Bacteroides clarus*	64.9	0.037
	*Porphyromonas asaccharolytica*	29.6	0.011		*Bifidobacterium bifidum*	56.1	0.025
**Post sort (differentially targeted)**							
**IgA Target in CD progressors**	*Bacteroides vulgatus*	43.9	0.010	**IgA Target in CD progressors**	*Enterobacter*	655	0.019
	*Clostridium sensu stricto*	28.5	0.010		*Blautia*	167	0.007
					*Clostridium sensu stricto*	24.6	0.040
	*Ruminococcus*	36.6	0.010				
					*Roseburia*	34	0.044
	*Actinomyces turicensis*	15.4	0.015				

### CD progressors have more bacteria coated with IgA, indicating a distinct humoral immune response in the intestines

**Fig. 2:**
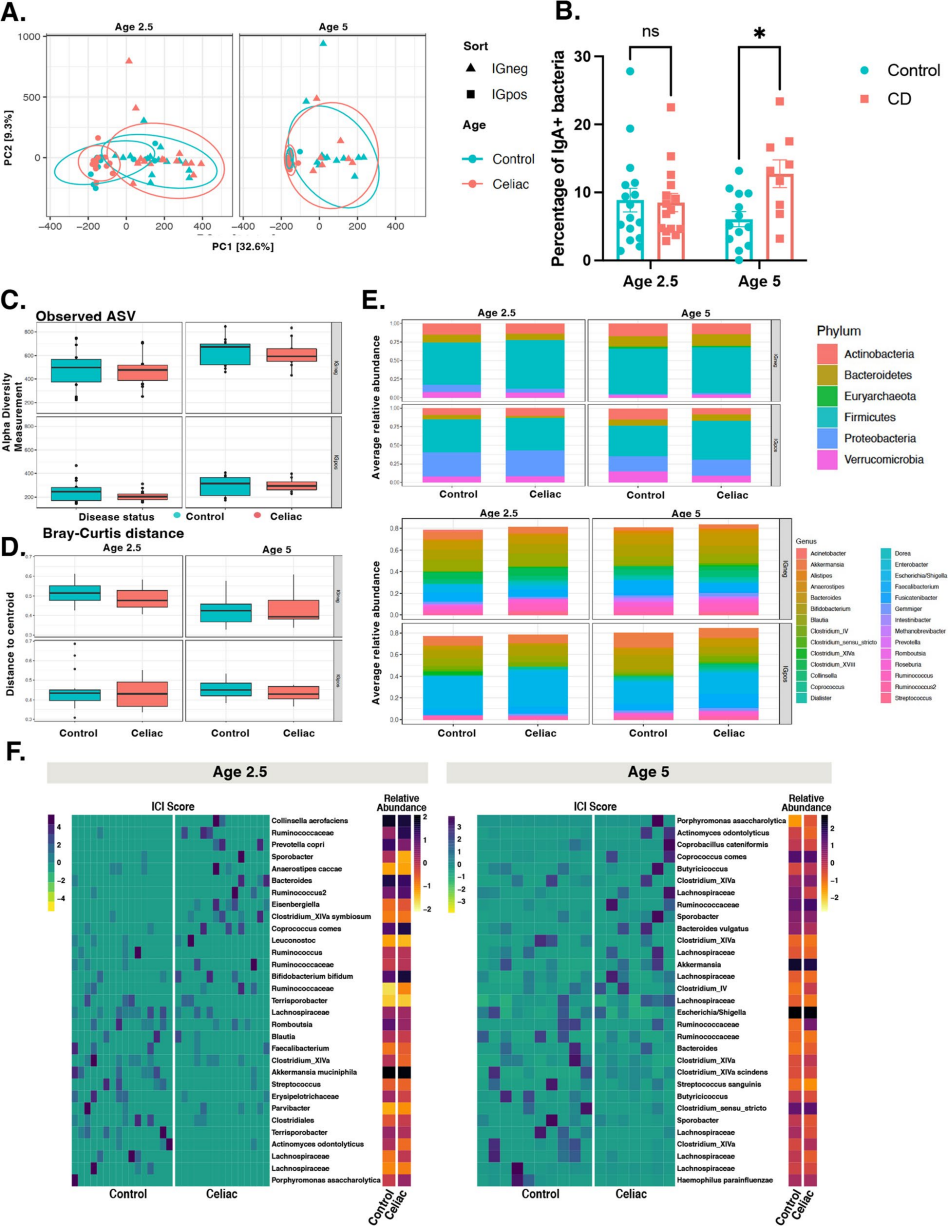
Gut microbiota of children developing CD have more IgA+ bacteria than controls at age 5. **A** Principal component analysis (PCA) of sample similarity/dissimilarity between IgA+ and IgA− microbiota in control (green), CD progressors (red) at age 2.5 (left), or age 5 (right). **B** Flow cytometry results for IgA + bacteria in fecal samples comparing CD progressors and healthy controls at ages 2.5 and 5. Indicated are mean ± SEM. Data were expressed as means±SEM. *p<0.05, **p<0.01, ***p<0.001. Statistical analysis was performed by using two-way ANOVA. **C** Box plots showing the comparison between CD progressors and healthy controls: the alpha diversity measured by observed for IgA+/IgA− microbiota at ages 2.5 (left panel) and 5 years old (right panel). Statistical analysis was performed using ANOVA. **D** Box plots showing the comparison between CD progressors and healthy controls: the beta diversity measured by Bray–Curtis dissimilarity for IgA+/IgA− microbiota at ages 2.5 (left panel) and 5 years old (right panel). Statistical analysis was performed using PERMANOVA. **E** Average relative abundance of IgA+/IgA− bacterial phylum (upper panel) or genera (lower panel) of greater than 1% abundance (proportion) between the gut microbiota of CD progressors and healthy controls at ages 2.5 (left panel) and 5 years old (right panel). Taxa average relative abundance>1%. Statistical analysis was performed using two-tailed *t* tests with Benjamini and Hochberg method to control the false discovery rate (FDR). **F** Heatmap of normalized IgA coating index (ICI) scores and relative abundance in 31 taxa for age 2.5 (left) and age 5 (right) in healthy and celiac disease progressors. Taxa with ICI values lower than 1 were discarded, except where ICI values for a particular taxon were (ICI_Control_ <1 and ICI_Celiac_ > 1) or (ICI_Control_ >1 and ICI_Celiac_ <1)

Immunoglobulin A (IgA) is the most abundant antibody isotype at mucosal surfaces and is a major mediator of intestinal immunity in humans [28]. IgA-sequencing (IgA-seq) combines bacterial cell sorting with high-throughput sequencing to identify distinct subsets of highly IgA-coated (IgA+) and non-coated microbiota (IgA−) [16, 17, 29, 30]. In this study, we used IgA-seq to determine the bacterial targets of IgA in the gut microbiota of CD progressors (*n*=15, age 2.5; *n*=9, age 5) compared to the matched healthy children (*n*=16, age 2.5; *n*=13, age 5). The flow chart and the gating strategy are described in Additional file 1: Figures S3A and S3B. PCA showed a clear separation between IgA+ and IgA− bacteria at age 2.5; however, no clear separation was observed between healthy and CD progressors’ samples at both ages (Fig. 2A). The flow cytometry analysis revealed that there was a two-fold increase of IgA+ bacteria in CD progressors (12.8%) compared to the controls (6.02%), specifically at age 5 (*n*=9–13, *p*=0.027, Fig. 2B). Overall, our data show that only a small fraction of the gut bacteria is coated by IgA in the first 5 years of gut microbiota development in both healthy controls and CD progressors. At age 5, CD progressors have more IgA+-coated bacteria compared to healthy controls indicating an altered humoral immune response in CD progressors.

### The targets of IgA are comprised of different bacteria in CD progressors

There are very few reports on the IgA response in early human gut microbiota development [30]. Thus, we first focused on the results obtained from healthy children. We identified 89 ASVs at age 2.5 and 37 ASVs at age 5 that were significantly different between IgA+ and IgA− samples in healthy controls (FDR<0.05, *p*<0.01; Additional file 1: Figure S3C, Additional file 2: Table S2). The top ASV targets of IgA in healthy subjects at age 2.5 were *Clostridium IV* and *Bifidobacterium*, and at the species level, *Bacteroides clarus*, *Catenibacterium mitsuokai*, and *Actinomyces odontolyticus. Clostridium IV* and *Gemmiger* were the top enriched ASVs, and *B. clarus*, *Bifidobacterium bifidum*, and *Clostridium sensu stricto* were the top enriched ASVs at the species level at age 5, indicating an immunoregulatory role of these bacteria in healthy children (Table 1). On the other hand, we identified 92 ASVs at age 2.5 and 5 ASVs at age 5 that were significantly different between IgA+ and IgA− samples in CD progressors (Additional file 2: Table S2). The top IgA-targeted taxa were reported in Table 1. CD progressors shared similar top IgA ASV targets with those in healthy subjects such as *Clostridium IV*, *Elizabethkingia*, and *B. bifidum* at ages 2.5 and *Clostridium IV*, *Brucella*, *Streptococcus*, *Elizabethkingia*, and *Pseudomonas* at age 5. On the other hand, we observed unique ASV targets of the IgA in CD progressors at age 2.5, including *Coprococcus comes*, *Clostridium sensu stricto*, *Leuconostoc*, *Bifidobacterium bifidum*, and *Actinomyces turicensis*.

Consistent with the presorting data, both alpha and beta diversity were comparable in post-sorting samples (IgA−, IgA +) (Fig. 2C, D). No difference was observed in the relative abundances of IgA+ or IgA− microbiotas at the phylum or genera level (Fig. 2E, Additional file 2: Table S3). On the other hand, we identified significant differences at the ASV level. At age 2.5, IgA-Seq identified 124 different ASVs between control IgA− samples and CD progressors’ IgA− samples. Likewise, we identified 132 different ASVs between control IgA+ samples and CD progressors’ IgA+ samples (FDR<0.05, Additional file 1: Figure S3DE, Additional file 2: Table S2). At age 5, 18 ASVs were different between control IgA− and CD IgA− samples, and 28 ASVs were different between CD IgA+ and control IgA+ samples (FDR<0.05, Additional file 1: Figure S3D, Additional file 2: Table S2). The top differential targets of the IgA+ response enriched in CD progressors were *Clostridium IV*, *Gemmiger*, and at species-level, *Dialister propionicifaciens*, *Bacteroides vulgatus*, and *Clostridium sensu stricto* at age 2.5. Moreover, *Dialister*, *Enterobacter*, *Clostridium XlVa*, *Roseburia*, *Bacteroides*, and at species-level *Dialister propionicifaciens*, *Clostridium sensu stricto*, ASVs were highly coated with IgA in CD groups at age 5.

In addition to the differences caused by altered gut microbiota composition, we also identified 53 ASVs at age 2.5 and 12 ASVs at age 5, in which abundances were comparable in the gut microbiota of CD progressors and healthy controls (presorting) but differentially targeted by the immune system (Additional file 2: Table S4). For example, *Bacteroides vulgatus*, *Clostridium sensu stricto*, (Additional file 1: Figure S3E), and *A. turicensis* ASVs at age 2.5 and *Enterobacter*, *Roseburia, Lactococcus*, and *Clostridium sensu stricto* ASVs at age 5 were comparable in their abundance; however, they were highly coated with IgA in CD progressors but not in controls (Additional file 1: Figure S3F, Figure S4A & B). Next, we calculated the IgA-coating index (ICI) score to quantify the IgA coating in healthy controls and CD progressors (Additional file 2: Table S5, Fig. 2F). We identified 34 taxa in healthy children and 22 taxa in CD progressors who had ICI scores>10 at age 2.5. Similarly, we identified 21 taxa in healthy controls and 19 taxa in CD progressors with ICI scores>10 at age 5 (Additional file 2: Table S5, Fig. 2F). Among these taxa, *Coprococcus comes* has an ICI score over 10 and is significantly increased in CD progressors. *Prevotella copri* and *Ruminococcaceae* taxa have an ICI score >10 in CD progressors at age 2.5 (Additional file 2: Table S5). *Actinomyces odontolyticus* taxon had an ICI score >10 and was significantly increased in CD progressors at age 5. Overall, these results indicate that not only gut microbiota composition but also the IgA response to gut microbiota is significantly altered in CD progressors.

### CD progressors have increased inflammatory cytokines and chemokines before diagnosis

**Fig. 3:**
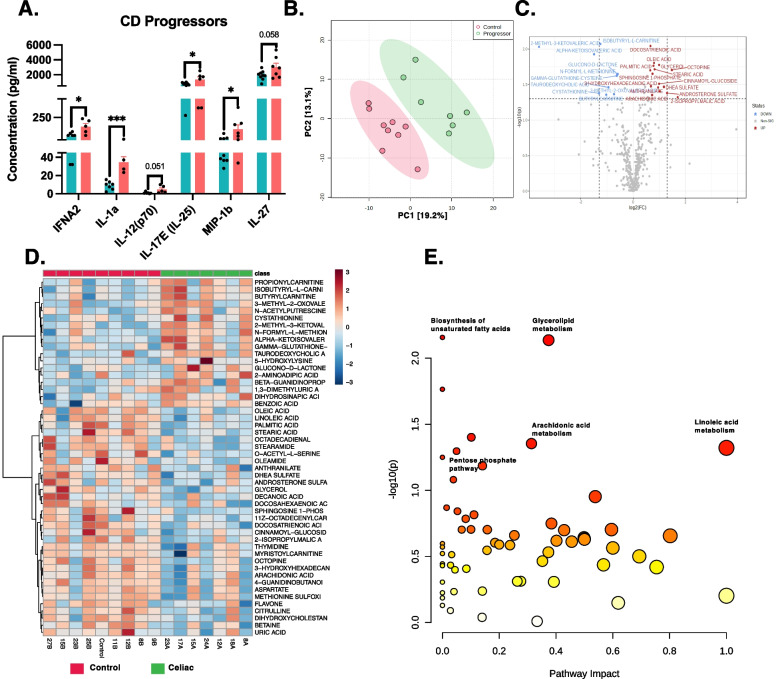
Plasma cytokines and metabolite levels alter in CD progressors. **A** Cytokine profile of the CD progressors (*n*=7) in comparison to healthy controls (*n*=9) at age 5 was assessed by Luminex. Data were expressed as means ±SEM. *p<0.05, **p<0.01, ***p<0.001. Statistical analysis was performed by a two-tailed, unpaired Student’s *t* test. **B** Partial least square-discrimination analysis (PLS-DA) of plasma metabolites for CD progressors (*n*=7) and controls (*n*=9). **C** Volcano plot of plasma metabolites of CD progressors vs healthy controls with fold change threshold (|log_2_ (FC)|>1.2) and *t* tests threshold (-log_10_(*p*)>0.1). The red dots represent metabolites above the threshold. Fold changes are log_2_ transformed, and *p* values are log_10_ transformed. **D** Heatmap showing 50 of the most altered metabolites between CD progressors and healthy controls. Each column represents an individual participant, and each row represents a metabolite. **E** The pathway analysis (combined results from powerful pathway enrichment analysis with pathway topology analysis) identify the most altered metabolic pathways between CD progressors and healthy controls. Pathway impact value (*x*) is calculated from pathway topology analysis. *p* is the original *p* value calculated from the enrichment analysis and depicted on a logarithmic scale

Our data showed that CD progressors have a distinct and stronger IgA response in the gut. To determine if this is consistent with systemic inflammation, we analyzed the cytokine profiles of seven CD progressors and nine healthy subjects at age 5 (Fig. 3A). Assessing 48 cytokines (Additional file 1: Figure S5A), we identified three proinflammatory cytokines (IFNA2, IL-1a, IL-17E/(IL25)) and a chemokine (MIP-1b) that were significantly increased in CD progressors before diagnosis. IL-27 and IL12(p70) also show a trend of an increase in CD progressors (Fig. 3A). The increase of these inflammatory cytokines in CD progressors might have a potential role in CD progression.

### Plasma metabolomic analysis reveals an inflammatory metabolic profile for CD progressors

To determine the early markers of CD progression in the plasma metabolome, we applied a targeted plasma metabolomics analysis. We used plasma samples obtained from seven CD progressors and nine matched healthy controls at age 5. In total, we identified 387 metabolites, and partial least squares-discriminant analysis (PLS-DA) showed a clear separation of plasma metabolites (Fig. 3B). Volcano plots show the most significantly altered metabolites (Fig. 3C, Additional file 2: Table S6). Specifically, 26 out of 387 metabolites were significantly altered (*p* < 0.05, Additional file 2: Table S5, Fig. 3D). The most altered metabolites (*p* < 0.01) were 2-Methyl-3-ketovaleric acid (FC=12.19) (Fig. 3D), TDCA (FC=1.92), Glucono-d-lactone (FC=1.51), and Isobutyryl-l-carnitine (FC=2.38), and all were increased in CD progressors. In addition, the anti-inflammatory metabolite, oleic acid (FC=0.57), was significantly decreased in CD progressors [31].

One of the most altered metabolites, TDCA, is a conjugated bile acid that is mainly produced by gut microbes, particularly *Clostridium XIVa* and *Clostridium XI*, with 7-α-dehydroxylation of taurocholic acid and cholic acid [32]. TDCA was previously shown to be a proinflammatory metabolite [33]. Moreover, this result is consistent with our microbiota analysis since we identified several *Clostridium XIVa* ASVs that were significantly more abundant in CD samples, especially at age 5 (FC=39.5, FDR=0.01). Furthermore, *Clostridium XIVa* ASVs were highly targeted by IgA in CD progressors (FC=305, FDR=0.006) (Additional file 1: Figure S5C). Using pathway analysis (Fig. 3F, Additional file 2: Table S7), we determined the functions related to these metabolites. Consistent with the previous reports, we showed that the pentose phosphate pathway (PPP) (raw *p*=0.05) was significantly altered in the CD progressors [9]; in addition to biosynthesis of unsaturated fatty acid (raw *p*=0.006), glycolipid metabolism (raw *p*=0.007), fatty acid elongation (raw *p*=0.017), galactose metabolism (raw *p*=0.039), arachidonic acid metabolism (raw *p*=0.044), and linoleic acid metabolism (raw *p*=0.047) pathways.

### TDCA treatment resulted in villous atrophy and stimulated inflammation in C57BL6/J mice

**Fig. 4:**
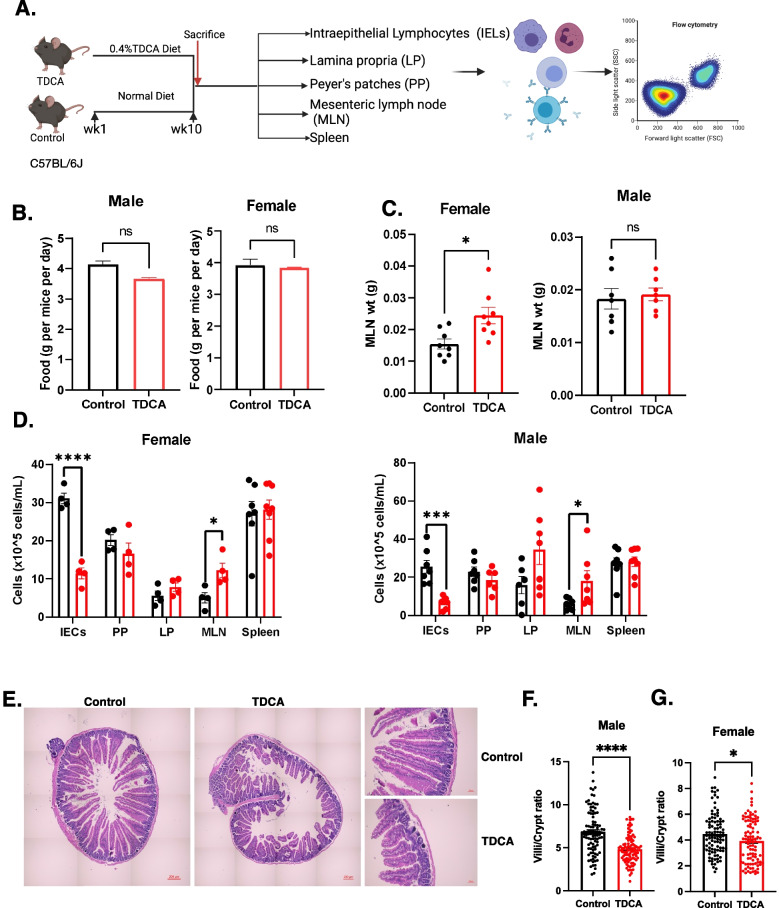
TDCA diet decreases villi/crypt ratio in the duodenum and changes T cell composition in different cell subsets. **A** Schematic overview of the experiments (*n*=8/group/sex). Mice were fed either a chow diet (control) or a chow diet containing 0.4% TDCA for 10 weeks. **B** Food intake of mice is represented as food intake per gram per mouse per cage. **C** Weight of the mesenteric lymph nodes (MLN). **D** Cell count was performed using a hemocytometer for each intestinal epithelium cell (IECs), Payer’s Patches (PP), Lamina Propria (LP), MLN, and spleen after isolation. Female (left panel) and male (right panel). **E** H&E images of duodenum sections of control and TDCA-treated female mice. Full image (left panel, scale =200 μm) and image at high magnification (right panel, scale =50 μm). **F** Villi/Crypt ratio of duodenum sections of control and TDCA-treated female mice (100 villi/crypt/group). **G** Villi/Crypt ratio of duodenum sections of control and TDCA-treated male mice (100villi/crypt/group)

To determine the effects of TDCA on the host, C57BL/6J male and female mice were fed either with standard chow or chow supplemented with TDCA (0.4% wt/wt) as previously described [33] for 10 weeks (Fig. 4A). We did not observe any differences in the food intake caused by TDCA, both in the male and female treatment groups (Fig. 4B). At the end of the treatment, we analyzed different cell subsets, including IELs, LPs, MLNs, PPs, and splenocytes. The MLNs of female mice fed with TDCA were found to be enlarged compared to those of female control mice and tended to enlarge in male mice (Fig. 4C). Both female and male mice treated with TDCA had a significantly higher number of cells in the MLNs, indicating inflammation in the small intestines (Fig. 4D). TDCA treatment also decreased the number of intestinal epithelium cells (IECs) in both female and male mice (Fig. 4D).

The main characteristics of CD are an increase in intraepithelial lymphocytes (IELs), partial and total villous atrophy, and crypt hyperplasia [34]. To determine the intestinal histological changes caused by TDCA treatment, duodenum sections of control and TDCA-treated mice were stained with H&E. Figure 4E shows the representative images of the histological changes in the duodenum section of the TDCA-fed mice compared to the control mice. We observed a significant decrease in villi/crypt ratio in both male and female mice (Fig. 4F, G). We also analyzed the ileum sections of control and TDCA mice. We observed distortion in crypt structure and partial and total villous atrophy caused by TDCA treatment in mice (Additional file 1: Figure S7A). TDCA treatment did not cause any significant difference in the villi length, crypt depth, and villi/crypt ratio in the ileum after 10 weeks of treatment (Additional file 1: Figure S7B). However, we observed a five-fold increase in the plasma cells in the lamina propria (Additional file 1: Figure S7C). These observations indicate that an increased microbiota-derived metabolite, TDCA, can cause villous atrophy in the duodenum of mice.

### TDCA treatment increases CD4+ T cell, NKG2D receptor expression, and decreases T-regulatory cells in vivo

**Fig. 5:**
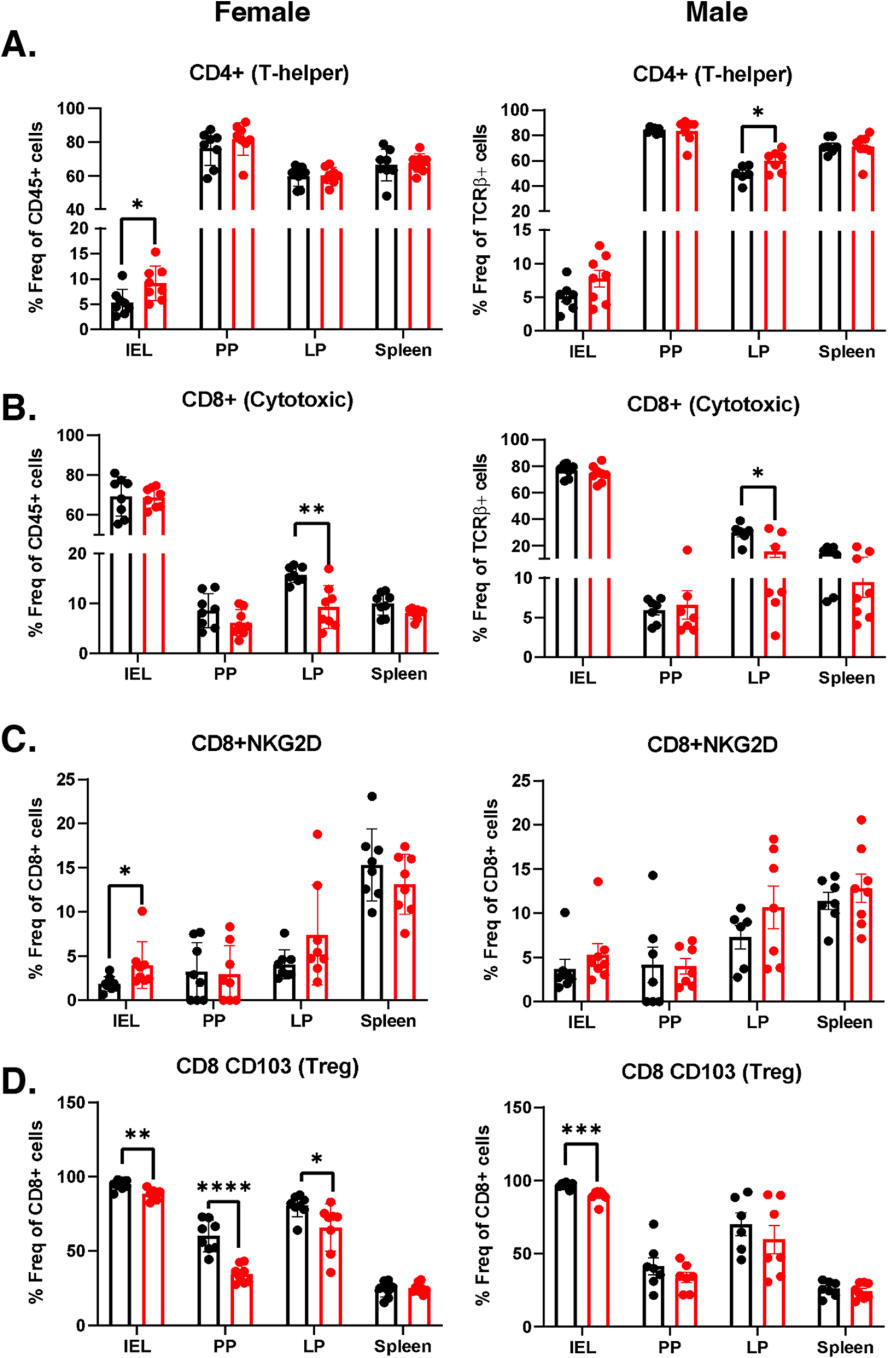
**A** CD4+ T cells as % of total TCRβ+ cells. **B** CD8+ T cells as % of total TCRβ+ cells. **C** CD8+ NKG2D+ T cells as % of total CD8+ cells. **D** CD8+ CD103+ T cells as % of total CD8+ cells in the IELs, PP, LP, and spleen of female (left panel) and male (right panel) mice. Control (black bar) and TDCA diet (red bar). Data were expressed as mean ± SEM. *p<0.05, **p <0.01,***p <0.01. Statistical analysis was performed by a two-tailed unpaired Student’s *t* test

figTo better understand the immune alterations stimulating the villous atrophy, we examined the different T cell and natural killer (NK) cell populations in the IELs, PPs, LPs, and spleen after 10 weeks of TDCA treatment. The gating strategy for determining all cell types has been described in Additional file 1: Figure S6. TDCA treatment induced a 2-fold increase in TCRβ+ cells in the PPs of both male and female mice (Additional file 1: Figure S7D). Furthermore, we identified a 2.1-fold increase in CD4+ T cells in the IELs of TDCA-treated female mice and observed a similar trend for the male mice. There was also a 25% increase in CD4+ T cells in LPs, specifically in TDCA-treated male mice (Fig. 5A). TDCA treatment did not change the CD8+ T cell population in the IEL, PP, and spleen but decreased in the LPs (Fig. 5B).

The NKG2D receptor is specifically expressed at the surface of all CD8+ αβT cells, γδT cells, and most NK cells [35] and plays a major role in the disruption of epithelium cells in CD pathogenesis by interacting with the MHC class I chain-related proteins A (MICA) protein [36]. MICA expression is increased in inflamed and stressed cells and serves as a ligand for the NKG2D receptor for immune cell activation. TDCA treatment caused a 2-fold increase in the NKG2D receptor on T cells in the IELs of the female mice, and a similar trend was observed in male mice (Additional file 1: Figure S7E). TDCA treatment also stimulated a 2-fold increase in the NKG2D expression on CD8+ T cells in IEL of female mice and a trend of increase for the male mice (Fig. 5C).

T-regulatory (Treg) cells play a key role in immune system homeostasis by immunosuppressing pathogenic T cells [37]. Treg function is impaired in CD patients [38]. CD8a+ TCRβ+ CD103+ cells, a novel subtype of CD8+ Treg cells, complement the function of CD4+ Foxp3+ Treg cells in the suppression of the immune response [39]. We analyzed both CD4+CD103+ and CD8+CD103+ Tregs. TDCA treatment decreased CD4+CD103+ T cells in IELs in both female (2.45-fold) and male mice (3-fold) (Additional file 1: Figure S7F). TDCA treatment also decreased CD8+CD103+ cells by 15.7% in IELs, 42.5% in PP, and 15.8% in LP as compared to female controls. We observed a decrease of 9.5% of CD8+CD103+ T cells in IELs of male mice with TDCA treatment (Fig. 5D).

**Fig. 6:**
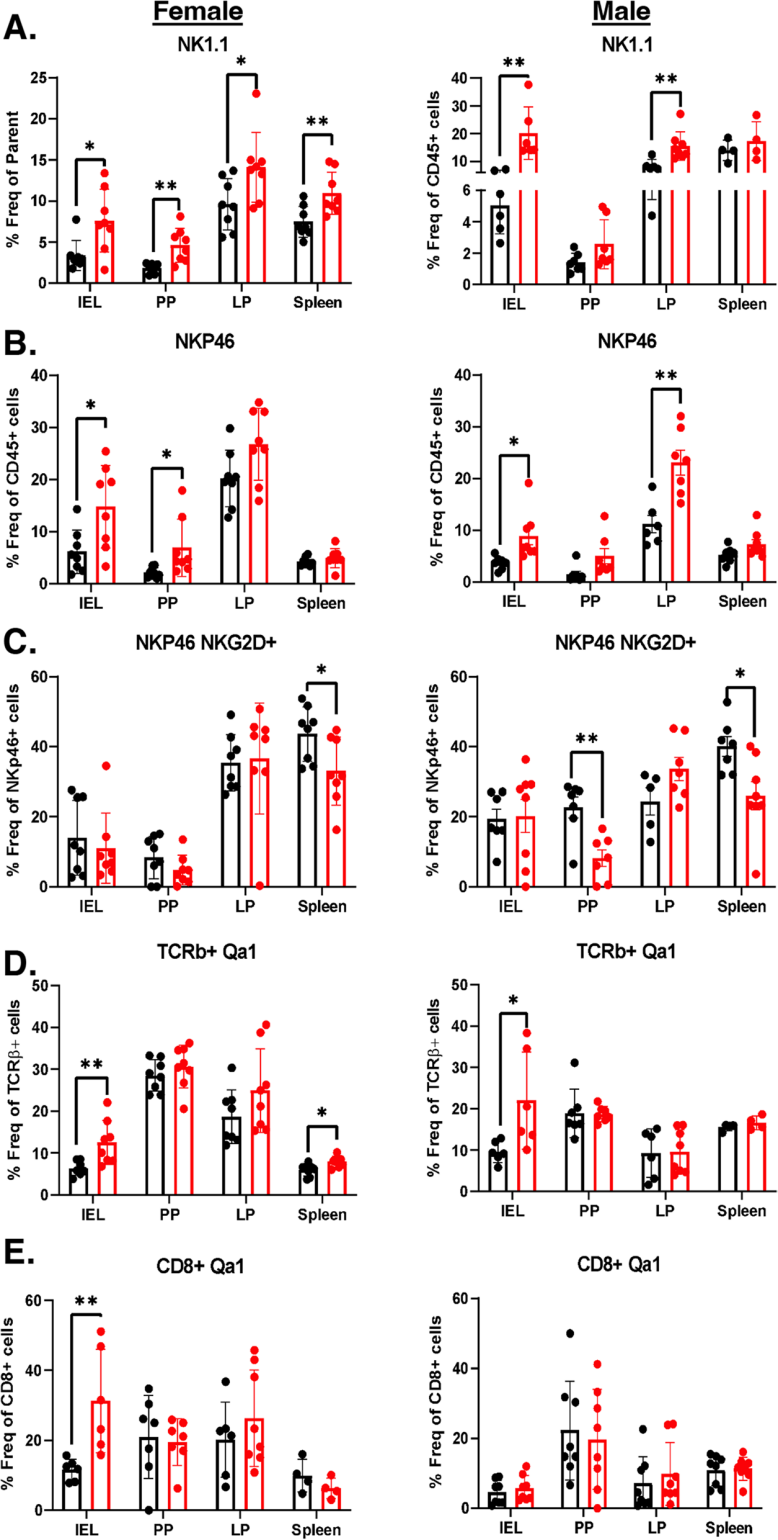
TDCA diet increases NK cells but decreases NK cells’ activity via increasing Qa-1 expression. Mice were fed either a chow diet (control) or a chow diet containing 0.4% TDCA for 10 weeks. **A** NK1.1+ cells as % of total CD45+ cells. **B** NKp46+ cells as % of total CD45+ cells. **C** NKp46+ NKG2D+ cells as % of total NKP46+ cells. **D** Qa-1+ cells as % of total TCRβ+ cells **E** Qa-1+ cells as % of total CD8+ cells in the IELs, PP, LP, and spleen of female (left panel) and male (right panel) mice. Data were expressed as mean ± SEM. *p<0.05, **p<0.01. Statistical analysis was performed by a two-tailed unpaired Student’s *t* test

TDCA treatment had a significant effect on the NK cell population in female mice. TDCA increased NK1.1+ cells in IEL (2.4-fold), PP (2.5-fold), LP (1.5-fold), and spleen (1.6-fold) (Fig. 6A). Likewise, TDCA-treated male mice had an increase in the NK1.1+ cells in IEL (4-fold) and LP (2-fold) (Fig. 6A). It also increased NKp46+ cells in IEL (2.4-fold) and PP (3.3-fold) in female mice and IEL (2.4-fold) and LP (2-fold) in male mice (Fig. 6B). Expression of two or more receptor-activating receptors (such as NKG2D, NKp46, NKp44, DNAM1, NKp80, 2B4, and CD16) are required to trigger the activation of naïve NK cells. Thus, we analyzed both NKG2D+ NKp46+ markers on NK cells. Notably, the NKG2D+ NKp46+ population decreased by 1.3-fold in female splenocytes, by 2.7-fold in PP, and by 1.5-fold in the splenocytes of male mice (Fig. 6C).

### TDCA treatment increases Qa-1 expression in cytotoxic T cells, potentially protecting T cells from lysis

TDCA treatment stimulated a 2-fold increase in Qa-1, a murine homolog of HLA-E, expression in the TCRβ+ cells in IELs in both female and male mice, and a 1.3-fold increase in splenocytes of female mice (Fig. 6D). It is also increased 3-fold on CD8+ T cells of IELs in male mice (Fig. 6E) and 1.2-fold on CD4+ T cells of splenocytes in female mice (Additional file 1: Fig. S7G). We also determined that the Qa-1 gene expression in the ileum tissue tended to increase in the ileum of both female (*p* = 0.069) and male mice (Additional file 1: Fig. S7H). A previous study showed that the increased expression of Qa-1 on T cells protects activated CD4+ T cells from lysis by a subset of NK cells and disrupts the CD8+ Treg response [40]. The decrease in Treg cell activity was observed in a reduction of IL-10 gene expression in the ileum tissue (Additional file 1: Fig. S7H). The reduction in the lysis of activated CD4+ T cells results in an increased number of autoreactive CD4+T cells. In addition, defective Qa-1-restricted T cell regulatory activity can result in autoimmunity [41]. Notably, in the DQ8-D^d^-Villin-IL-15tg mouse model of CD, Qa-1 expression increases in the intestinal epithelium after gluten ingestion and is reduced when mice are fed with a gluten-free diet [42], indicating an important link between Qa-1 and CD onset.

## Discussion

The gut microbiome plays a significant role in digestion, immune modulation, gluten metabolism, and the maintenance of gut permeability. Several previous reports have shown alterations in the gut microbiome composition in CD patients [8–13, 43, 44]. While these studies provided important resources, it is still unknown whether these microbial changes are resulting from the disease onset or if they have a causal role in CD onset. To identify a causal link between gut microbiota and CD onset, we completed a comprehensive study and determined the alterations in the gut microbiome, IgA response, plasma metabolome, and cytokine profile of CD progressors compared to healthy children. Using 16S sequencing, we identified significant differences at the ASV level, which is more informative than alterations at the phylum or genera level. For example, we observed a significant decrease in the abundance of some ASVs at age 2.5 that are members of the *Bacteroides* genus [45]. Anti-inflammatory species, including *B. uniformis* [9], and *B. stercoris* [8], are decreased in CD progressors as compared to healthy subjects. These results are consistent with the previous reports showing a decrease in CD progressors and CD patients [11]. Moreover, taxa including *Prevotella* and *Holdemanella* were decreased in CD progressors at age 5, and these genera were previously reported to decrease in CD patients [11, 46]. We observed an increase in *Dialister* ASVs in CD progressors at both ages, which is also consistent with the previous reports [11].

Planer et al. described mucosal IgA response progression in healthy US twins during two postnatal years [30]. We used a similar approach to investigate the gut IgA immune development towards healthy and CD states; focusing on the development of the IgA response during gut microbiota maturation. Flow cytometry analyses showed that the IgA response is highly selective, and only a small fraction of the gut microbiota is highly coated with IgA. Notably, the percentage of IgA+ bacteria was higher in CD progressors compared with healthy controls at age 5. A reduction of secretory IgA (sIgA) in infant (4–6 months) fecal samples in CD progressors [10] was previously reported. In contrast, we identified a two-fold increase in the number of IgA-coated bacteria in CD progressors at age 5. It is important to note that the sampling ages are different in these two cohorts. In our study, we identified significant differences between the IgA+ microbiota of controls and CD progressors (Fig. 2E). Our analysis revealed 133 ASVs at age 2.5 and 29ASVs at age 5 that were highly IgA coated in the CD progressors. Among those, ASVs, *Coprococcus comes*, and *Bifidobacterium bifidum* at age 2.5 and *Clostridium lV* at age both ages were identified as the top targets of the mucosal immune response in CD progressors. These taxa are regarded as gut barrier-protective commensals, as reported previously [47]. Among these bacteria, *C. comes* recently been identified as the main IgA target in the human colon [48], and *Clostridium* species can utilize large amounts of nutrients that cannot be digested by the host and produces short-chain fatty acids (SCFAs) that play a key role in intestinal homeostasis. *Actinomyces odontolyticus*, *Coprococcus comes*, *Ruminococcaceae*, and *Prevotella copri* taxa had an ICI score >10 in CD progressors. Among these, *Coprocccus comes* and *Ruminococcaceae* are butyrate producers and are negatively associated with inflammation [49, 50]. However, *Actinomyces odontolyticus* and *Prevotella copri* are reported to be inflammatory [51–53].

We identified some taxa whose abundance was comparable to their presorting abundance but was specifically targeted in CD progressors. Among these, *B. vulgatus* [54] and *Clostridium sensu stricto* were previously reported to be associated with CD onset [55]. On the other hand, *Bifidobacterium bifidum* is specifically targeted by IgA in healthy children at age 5 and had an ICI score >10 at both ages. Likewise, we identified an ASV of *Bifidobacterium* that is specifically targeted in healthy controls at age 2.5. *Bifidobacterium* species can stimulate the production of IgA in the intestines [56, 57] and are known to regulate immune response by inducing dendritic cells and Treg cells [58]. These results suggest that *Bifidobacterium* species may have an immunoregulatory function in healthy individuals that is lost in the CD progressors.

Although the hallmark of CD is an intestinal inflammation, the disease affects different tissues. To determine the systemic effects of CD onset, we measured the levels of 48 different cytokines, chemokines, and growth factors. Three proinflammatory cytokines and one chemokine were increased in the CD progressors. Among these, IL-12, IL1a, and IFNA2 cytokines were previously shown to increase in CD patients and decrease with a gluten-free diet (GFD) [59, 60]. IL-12 (p70) increased in children with CD [61]. Likewise, consistent with our results, MIP-1b/CCl4 chemokine increased in non-treated CD patients [61]. Among these, IL-17E (IL-25) is a “barrier cytokine” that plays a key role in regulating autoimmune processes via T_H_17 cells and maintaining homeostasis by alarming the immune cells about tissue injury [62]. Overall, our findings indicate the presence of an increased inflammatory response before the diagnosis.

Our analysis revealed that plasma metabolites were significantly altered prior to diagnosis in CD progressors. Pathway analysis for plasma metabolites identified several pathways, including the pentose phosphate pathway (PPP), biosynthesis of unsaturated fatty acid, glycolipid metabolism, fatty acid elongation, galactose metabolism, arachidonic acid metabolism, and linoleic acid metabolism pathways. A recent study on fecal metabolome in CD progressors showed that PPP is one of the most significantly increased pathways [9]. PPP is also one of the significantly altered pathways in our analysis. This pathway stimulates the formation of NADPH as an antioxidant by regenerating reduced glutathione and neutralizing the reactive oxygen intermediates (ROI), thereby controlling cell inflammation [63]. Pathways, including unsaturated fatty acid synthesis and fatty acid elongation, are reported to increase inflammation through activation of nuclear factor kappa B (NF-κB) and Toll-like receptor-4 (TLR-4) signaling [64, 65]. Moreover, an increase in galactose metabolism induces galactose production, which is reported to increase oxidative stress and gut dysbiosis [66].

TDCA, the significantly altered plasma metabolite in CD progressors, is a conjugated bile acid produced mainly by gut bacteria, particularly *Clostridium XIVa* and *Clostridium XI* [32]. Our data suggest that TDCA is potentially secondary to some *Clostridium XIVa* taxa that had increased abundance in CD progressors at age 5. In this study, we showed that chronic TDCA exposure is sufficient to mimic CD characteristics. TDCA treatment causes villous atrophy in C57B6/J mice, decreasing villi/crypt ratio in the duodenum and increasing CD4+ T cells in the IEL of the female and LP of the male mice. These are the main phenotypic changes reported during CD progression [67]. Thus, our findings show that TDCA, a microbiota-derived metabolite, is potentially linked to the CD progression-related alterations in the small intestines.

In CD, CD4+ T cells secrete several cytokines, increase T cell expansion, and participate in the killing of intestinal epithelial cells via the IELs [68]. Previous studies have shown that T cell-mediated killing of intestinal epithelium cells in CD is regulated by NKG2D receptor expression on a set of immune cells and NKG2D receptor’s interaction with MICA protein [36]. Here, we show that TDCA treatment induces the expression of NKG2D on T cells and on CD8+ αβT cells in IELs of female mice. In addition, we also identified a decrease in CD8+ CD103+ Treg cells. CD8+ Treg cells lyse effector T cells via perforin and other cytokines [69]. Thus, TDCA treatment stimulated a decrease in CD8+ Treg cells, potentially contributing to increased inflammation via increased effector T cells in the small intestines of the mice. We also show that TDCA treatment reduces NK cell activation by downregulating the surface expression of NKG2D/NKp46 receptors in the splenocytes of both male and female mice and PP of the male mice. This is also relevant to CD onset because active (NKp44/NKp46 double-positive) NK cells, which are responsible for the enhanced production of granzyme B, are reduced in active CD patients [70].

A subset of CD8+ Treg cells recognizes Qa-1, which is essential for maintaining self-tolerance [71]. Qa-1 inhibits NK cell-mediated autoreactive T cell killing via suppressing CD8+CD103+ Treg cells. Engagement of Qa-1 with the NKG2A receptor induces inhibitory signals for CD8+ Treg cells as well as NK cells, and this can increase autoimmune disease risk as shown in experimental autoimmune encephalomyelitis [72]. Here, we observed an increase in Qa-1 expression, a decrease in CD8+ Treg cells, and suppression of NK cell activation altogether, resulting in accelerated inflammation in the small intestines of the TDCA-treated mice. Currently, the only way to treat CD is strict adherence to a GFD, but 20% of patients do not respond to GFD and continue to have persistent or recurrent symptoms [73]. CD permanently reshapes intestinal immunity, and alterations of TCRγδ+ intraepithelial lymphocytes, in particular, may underlie non-responsiveness to the GFD [74]. Our findings suggest that the inflammatory nature of the CD progressors’ gut microbiota, especially the increased TDCA, is potentially one of the early key components of intestinal inflammation in CD. The proinflammatory factors identified in this study have the potential to trigger local and systemic inflammation independent of the diet and may explain a failure to respond to GFD in some patients.

## Conclusion

**Fig. 7:**
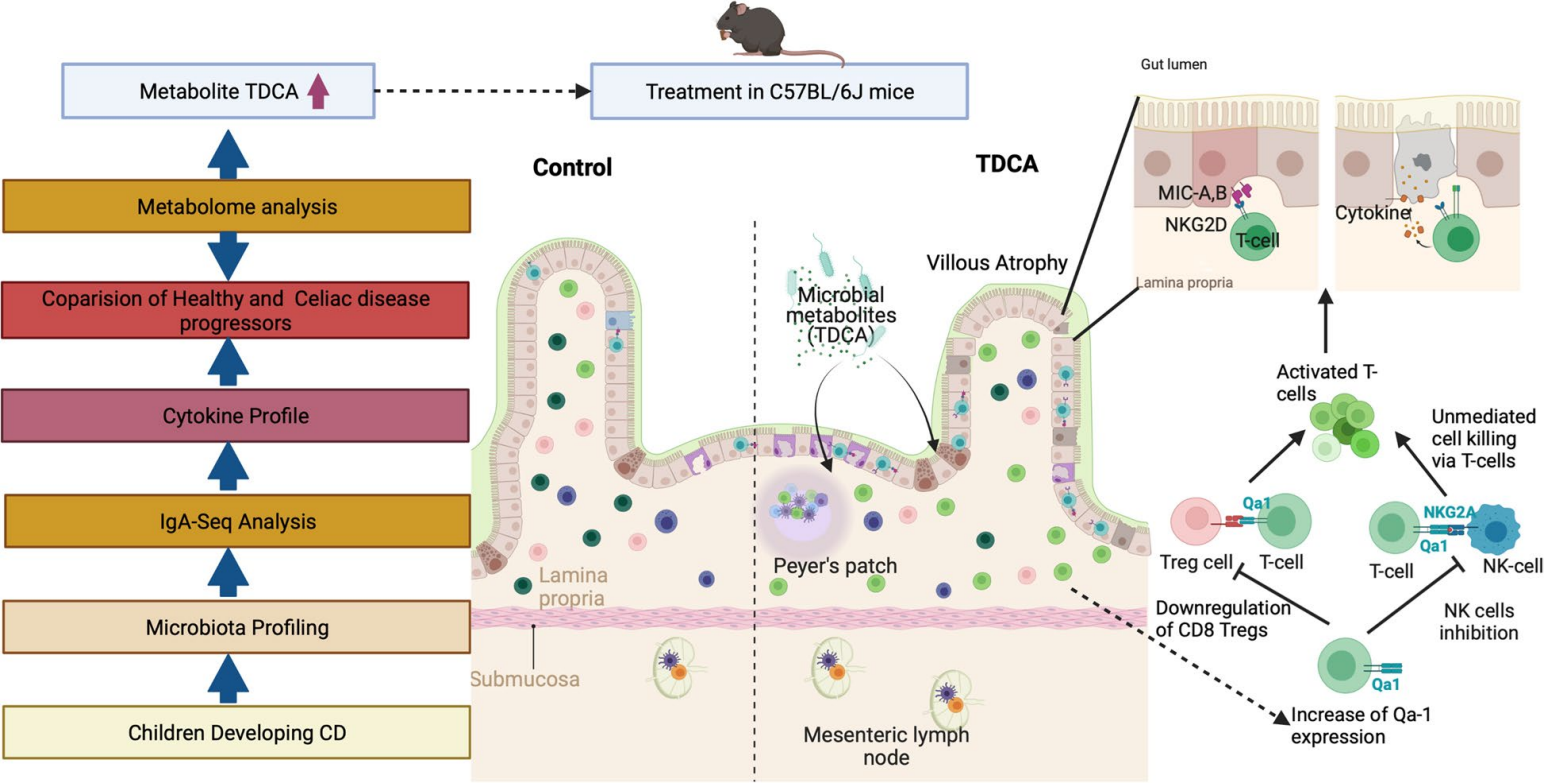
The experimental approach and the proposed mechanism of TDCA action on immune cells causing villous atrophy (Biorender.com)

Taken together, our findings identified a distinct gut microbiota, IgA response, plasma metabolome, and cytokine profile for the CD progressors before diagnosis. Further, we establish a potential link between TDCA, a metabolite specifically produced by gut microbes, and intestinal inflammation (Fig. 7). TDCA has the potential to serve as an early diagnostic marker of the disease, and more importantly, targeting TDCA-producing bacteria early in life could be an approach to manage CD. Understanding the role of the gut microbiota and its products, specifically TDCA, in CD onset has the potential to open novel avenues to understand disease pathogenesis and reveal new preventive and treatment models.

### Limitations

One of the main limitations of our study is the relatively small sample size. Age, sex, HLA subtype, and breast-feeding were shown to affect gut microbiota composition. Therefore, we matched healthy subjects and CD progressors’ samples based on these criteria, which significantly limited the sample size. Similar studies are needed to confirm our results using a larger sample size. In addition, the collection of the samples from a Swedish cohort makes it difficult to determine whether these alterations are specific to CD progressors in Sweden or indicate a universal mechanism/alteration related to gut microbiota and plasma metabolome. In addition, while we were able to identify significant differences in the species level using 16S sequencing, shotgun sequencing would identify more significant differences with high taxonomic resolution [75]. Lastly, we were able to show that TDCA treatment causes villous atrophy in C57BL/6J mice but this is not a model for CD progression.

## Supplementary Information

**Additional file 1: Figure S1.** Flow chart describing CD progressors demographics and sample size. **Figure S2.** Violin plot representation of most enriched genus/species in CD progressors compared to healthy controls. A. Fold change in ASVs at age 2.5 CD progressors (left panel, n=15) and healthy controls (right panel, n=16). B. Fold change in ASVs at age 5 in CD progressors (left panel, n=10) and healthy controls (right panel, n=13). **Figure S3.** Gating strategy for IgA sequencing and analysis. A. Schematic overview of IgA-based fecal bacteria separation combined with 16S rRNA gene sequencing (IgA-seq) for stool samples from CD progressors and healthy controls. MACS: Magnetic-activated cell separation. B. Gating strategy for the isolation of IgA-/+ bacteria from the CD progressors and healthy controls’ fecal samples. C. Empirical Bayes quasi-likelihood F-tests analysis for comparing IgA-coated and non-coated gut microbiota ASVs in healthy controls (upper row) and CD progressors (lower row) at ages 2.5, and 5. Frequency: number of ASVs. FDR: False Discovery Rate. D. Empirical Bayes quasi-likelihood F-tests analysis for the comparisons of IgA-coated or non-coated gut microbiota ASVs between CD progressors and healthy controls (upper row: age 2.5 years old; lower row: age 5 years old). F. Box plots showing representative ASVs in which abundances were similar in the gut microbiota (presorting samples) but differently targeted by IgA at age 2.5. E. Violin plots showing representative ASVs in which abundances were similar in the gut microbiota (presorting samples) but differently targeted by IgA at age 5. **Figure S4.** Heat Map showing IgA target in CD progressors and healthy subjects. A. Heat map showing the relative abundance of the top ASVs significantly different between IgA+ and IgA- samples of CD progressors and healthy controls (ASVs=51, selected based on p-value) at age 2.5 B. at age 5. Each column represents an individual participant and each row represents an ASV. **Figure S5.** The cytokine and plasma metabolome profiles of CD progressors and CD patients. A. Comparison of all 48 cytokines analyzed in plasma samples obtained from CD progressors (n=10) and healthy controls (n=10) at age 5. Data were expressed as means ±SEM. *p<0.05, **p<0.01, ***p<0.001. Statistical analysis was performed by a two-tailed, unpaired student’s t-test. B. Violin plots showing the representative *Clostridium XIVa* bacteria abundance between CD progressors and healthy controls (Left: before separation by IgA coating, Right: in IgA+ bacteria). **Figure S6.** Gating Strategy for the Flow cytometry analysis. Strategy 1- Gating strategy for NK1.1 and Qa-1 expression in TCRβ+ cells. Strategy 2- Gating strategy for CD8, CD4, NKG2D, CD103, and NKp46. **Figure S7.** TDCA diet induces changes in T-cell composition in different cell subsets. A. H&E images of ileum tissue sections of control and TDCA treated female mice. Full image (upper panel) and image at high magnification (lower panel). Scale =20μm. B. Villi/ Crypt ratio in ileum tissue sections of control and the TDCA treated female mice. C. Number of plasma cells in the lamina propria of the ileum section of female mice. D. TCRβ+ cells as % of total CD45+ cells. E. NKG2D+ cells as % of total TCRβ+ CD45+ cells. F. CD103+ cells as % of total CD4+ cells. G. Qa-1+ cells as % of total CD4+ cells in the IELs, PP, LP, and spleen of female (left panel) and male (right panel) mice. H Relative gene expression of Qa-1 and IL-10 in the ileum tissue analyzed using qPCR. Female (left panel) and male (right panel) mice after 10 weeks of TDCA treatment compared to controls. Data were expressed as mean ± SEM. *p<0.05, **p <0.01, ***p<0.001. Statistical analysis was performed by a two-tailed unpaired student’s t-test.

**Additional file 2:** **Table S1. **Detailed Cohort and Fecal Sample Information copy. **Table S2. **Complete 16S Taxonomy Data and Analysis. **Table S3.** Relative Abundance Analysis. **Table S4.** IgA Targeted ASVs in CD Progressors at 2.5 and 5. **Table S5.** Calculated ICI scores for each taxon. **Table S6.** Significantly Altered Plasma Metabolites. **Table S7.** Metabolic Pathway Pearson Correlation. **Table S8.** List of Flow cytometry antibodies.

## Data Availability

All ASV-related data analyzed in this study are included in this published article (Additional file 2: Tables S2). The 16S rRNA gene sequencing raw data generated in this study is available through the NCBI Sequence Read Archive Bioproject PRJNA631001. The plasma metabolomics data are included in this published article (See Additional file 2: Table S8). The gut microbiome analysis codes generated in this study are available at this link: https://github.com/altindislab/celiac-gut-microbiome.
