# Supplementary figures and images for: Dynamics of the gut microbiome, IgA response, and plasma metabolome in the development of pediatric celiac disease
Source: Microbiome. 2023 Jan 13;11:9. doi: 10.1186/s40168-022-01429-2 (PMC9840338; doi:10.1186/s40168-022-01429-2)

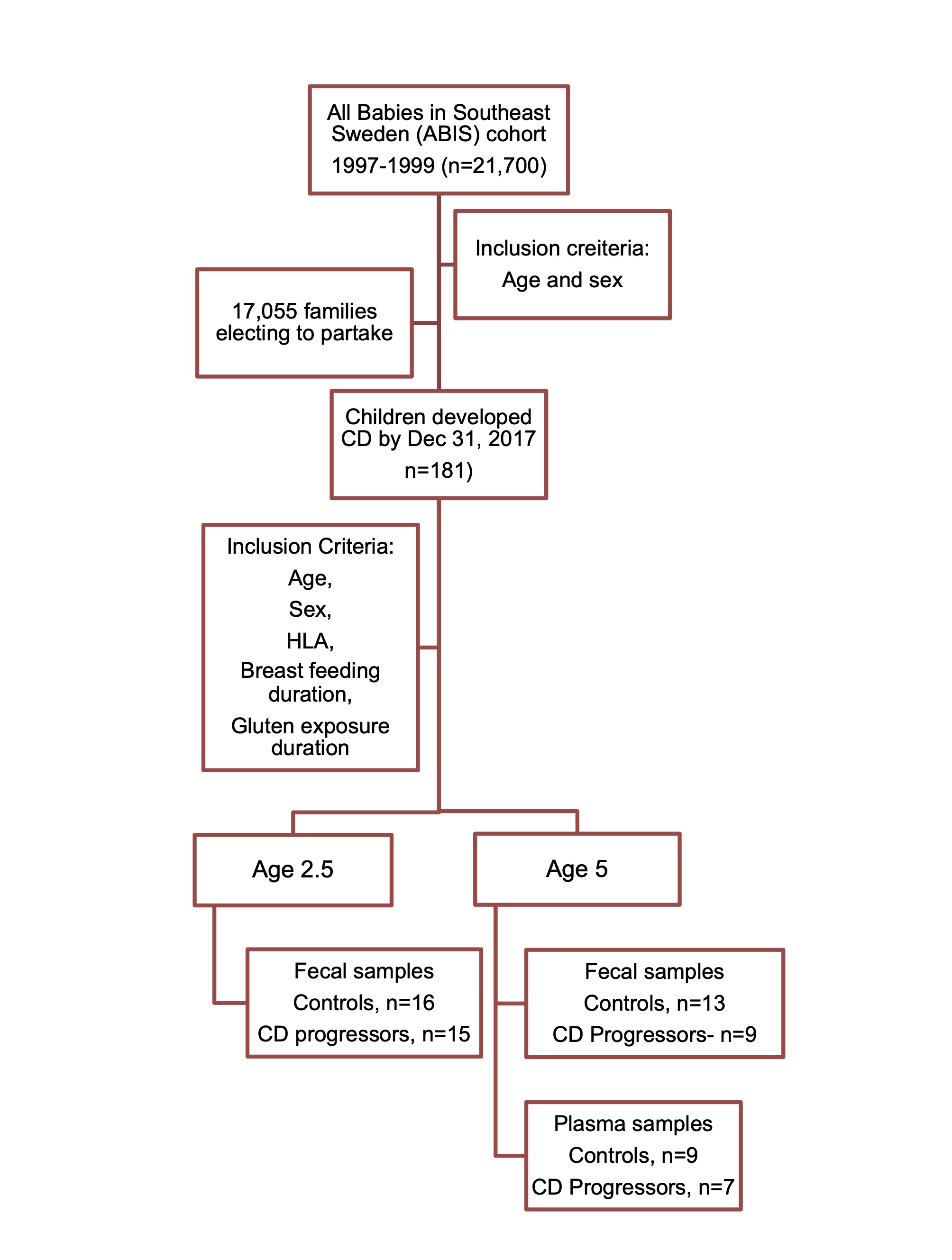

Supplement: Supplementary file 2 — Additional file 1: Figure S1. Flow chart describing CD progressors demographics and sample size. Figure S2. Violin plot representation of most enriched genus/species in CD progressors compared to healthy controls. A. Fold change in ASVs at age 2.5 CD progressors (left panel, n=15) and healthy controls (right panel, n=16). B. Fold change in ASVs at age 5 in CD progressors (left panel, n=10) and healthy controls (right panel, n=13). Figure S3. Gating strategy for IgA sequencing and analysis. A. Schematic overview of IgA-based fecal bacteria separation combined with 16S rRNA gene sequencing (IgA-seq) for stool samples from CD progressors and healthy controls. MACS: Magnetic-activated cell separation. B. Gating strategy for the isolation of IgA-/+ bacteria from the CD progressors and healthy controls’ fecal samples. C. Empirical Bayes quasi-likelihood F-tests analysis for comparing IgA-coated and non-coated gut microbiota ASVs in healthy controls (upper row) and CD progressors (lower row) at ages 2.5, and 5. Frequency: number of ASVs. FDR: False Discovery Rate. D. Empirical Bayes quasi-likelihood F-tests analysis for the comparisons of IgA-coated or non-coated gut microbiota ASVs between CD progressors and healthy controls (upper row: age 2.5 years old; lower row: age 5 years old). F. Box plots showing representative ASVs in which abundances were similar in the gut microbiota (presorting samples) but differently targeted by IgA at age 2.5. E. Violin plots showing representative ASVs in which abundances were similar in the gut microbiota (presorting samples) but differently targeted by IgA at age 5. Figure S4. Heat Map showing IgA target in CD progressors and healthy subjects. A. Heat map showing the relative abundance of the top ASVs significantly different between IgA+ and IgA- samples of CD progressors and healthy controls (ASVs=51, selected based on p-value) at age 2.5 B. at age 5. Each column represents an individual participant and each row represents an ASV. Figur [file 40168_2022_1429_MOESM1_ESM.zip › Figure S1.jpg]

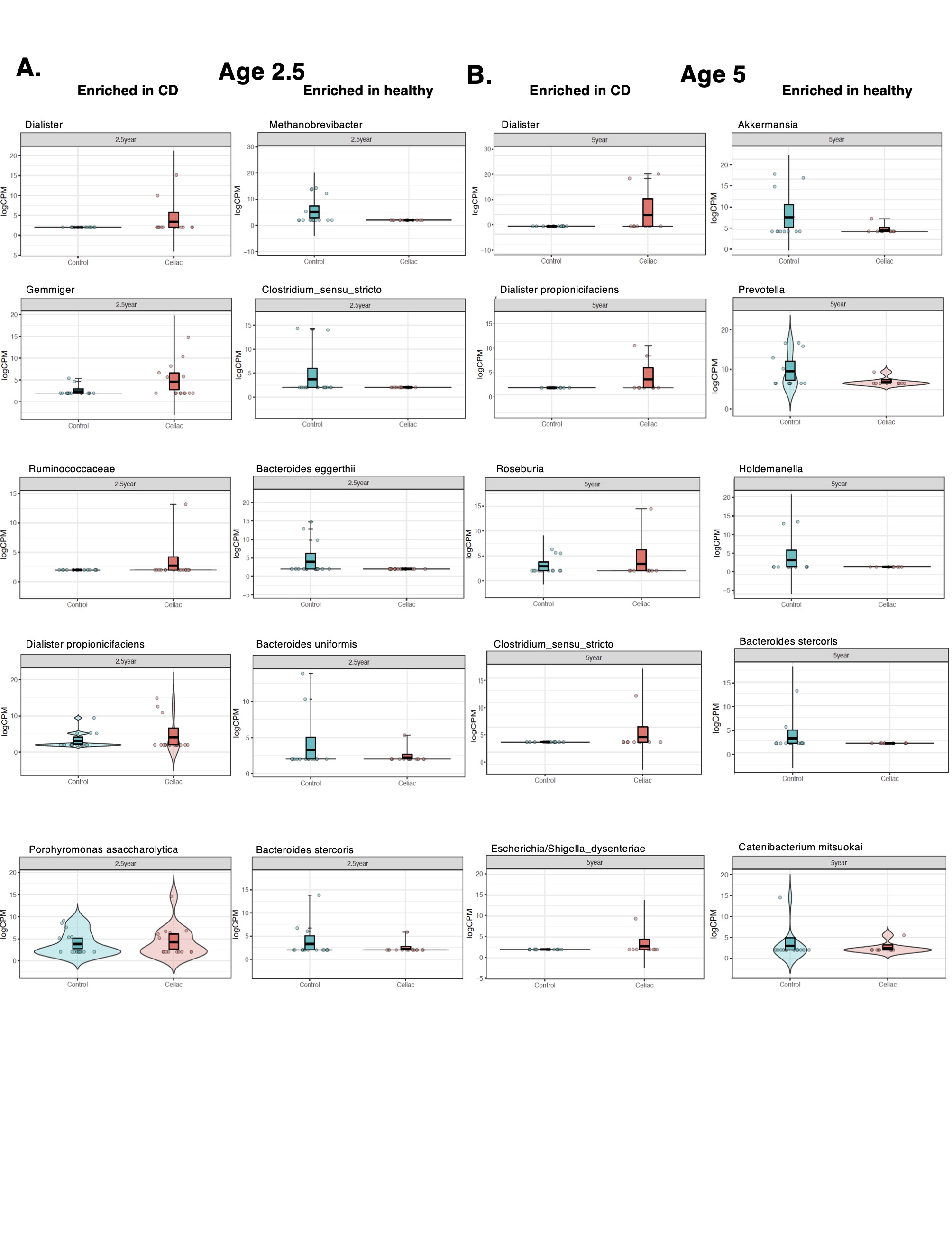

Supplement: Supplementary file 2 — Additional file 1: Figure S1. Flow chart describing CD progressors demographics and sample size. Figure S2. Violin plot representation of most enriched genus/species in CD progressors compared to healthy controls. A. Fold change in ASVs at age 2.5 CD progressors (left panel, n=15) and healthy controls (right panel, n=16). B. Fold change in ASVs at age 5 in CD progressors (left panel, n=10) and healthy controls (right panel, n=13). Figure S3. Gating strategy for IgA sequencing and analysis. A. Schematic overview of IgA-based fecal bacteria separation combined with 16S rRNA gene sequencing (IgA-seq) for stool samples from CD progressors and healthy controls. MACS: Magnetic-activated cell separation. B. Gating strategy for the isolation of IgA-/+ bacteria from the CD progressors and healthy controls’ fecal samples. C. Empirical Bayes quasi-likelihood F-tests analysis for comparing IgA-coated and non-coated gut microbiota ASVs in healthy controls (upper row) and CD progressors (lower row) at ages 2.5, and 5. Frequency: number of ASVs. FDR: False Discovery Rate. D. Empirical Bayes quasi-likelihood F-tests analysis for the comparisons of IgA-coated or non-coated gut microbiota ASVs between CD progressors and healthy controls (upper row: age 2.5 years old; lower row: age 5 years old). F. Box plots showing representative ASVs in which abundances were similar in the gut microbiota (presorting samples) but differently targeted by IgA at age 2.5. E. Violin plots showing representative ASVs in which abundances were similar in the gut microbiota (presorting samples) but differently targeted by IgA at age 5. Figure S4. Heat Map showing IgA target in CD progressors and healthy subjects. A. Heat map showing the relative abundance of the top ASVs significantly different between IgA+ and IgA- samples of CD progressors and healthy controls (ASVs=51, selected based on p-value) at age 2.5 B. at age 5. Each column represents an individual participant and each row represents an ASV. Figur [file 40168_2022_1429_MOESM1_ESM.zip › Figure S2.jpg]

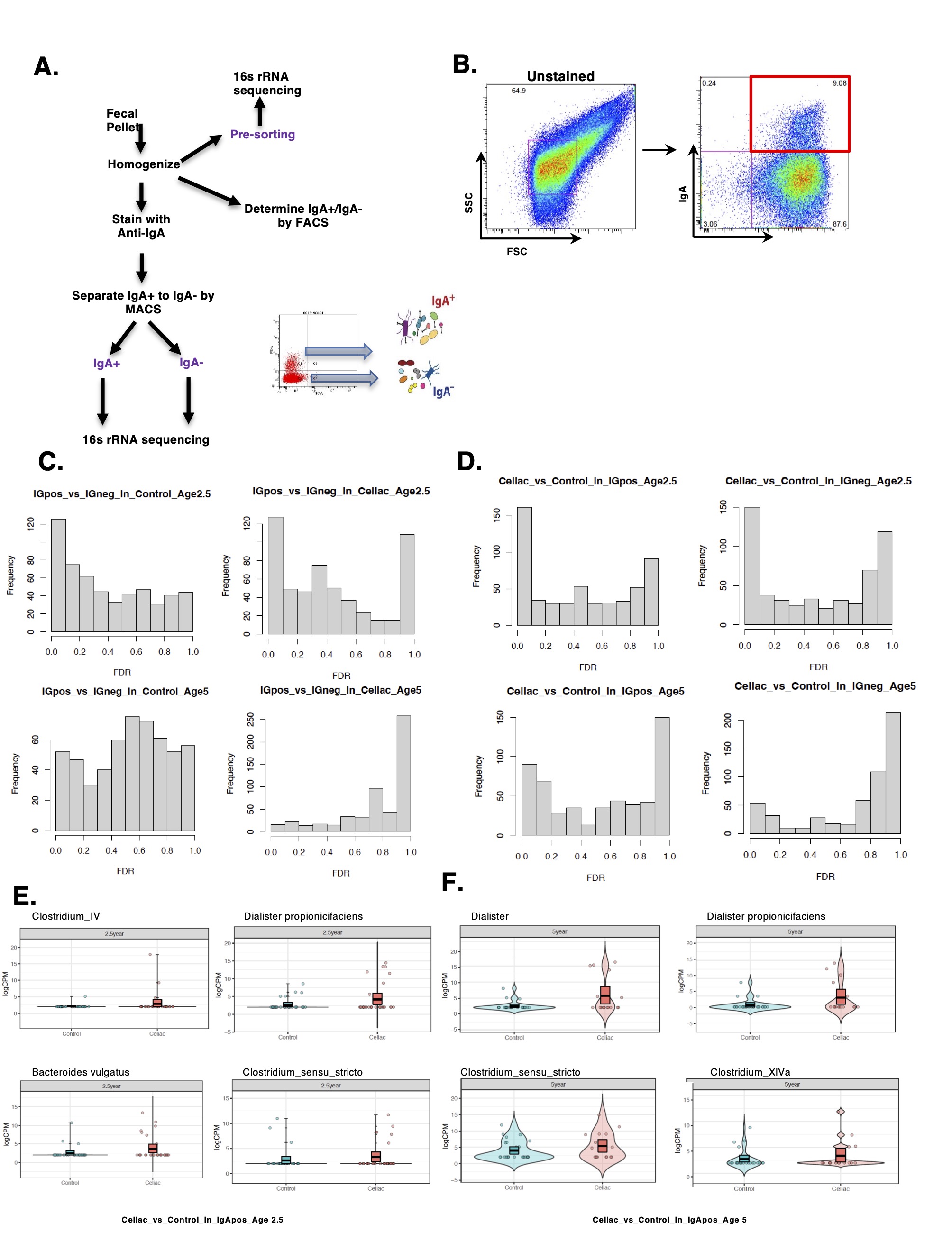

Supplement: Supplementary file 2 — Additional file 1: Figure S1. Flow chart describing CD progressors demographics and sample size. Figure S2. Violin plot representation of most enriched genus/species in CD progressors compared to healthy controls. A. Fold change in ASVs at age 2.5 CD progressors (left panel, n=15) and healthy controls (right panel, n=16). B. Fold change in ASVs at age 5 in CD progressors (left panel, n=10) and healthy controls (right panel, n=13). Figure S3. Gating strategy for IgA sequencing and analysis. A. Schematic overview of IgA-based fecal bacteria separation combined with 16S rRNA gene sequencing (IgA-seq) for stool samples from CD progressors and healthy controls. MACS: Magnetic-activated cell separation. B. Gating strategy for the isolation of IgA-/+ bacteria from the CD progressors and healthy controls’ fecal samples. C. Empirical Bayes quasi-likelihood F-tests analysis for comparing IgA-coated and non-coated gut microbiota ASVs in healthy controls (upper row) and CD progressors (lower row) at ages 2.5, and 5. Frequency: number of ASVs. FDR: False Discovery Rate. D. Empirical Bayes quasi-likelihood F-tests analysis for the comparisons of IgA-coated or non-coated gut microbiota ASVs between CD progressors and healthy controls (upper row: age 2.5 years old; lower row: age 5 years old). F. Box plots showing representative ASVs in which abundances were similar in the gut microbiota (presorting samples) but differently targeted by IgA at age 2.5. E. Violin plots showing representative ASVs in which abundances were similar in the gut microbiota (presorting samples) but differently targeted by IgA at age 5. Figure S4. Heat Map showing IgA target in CD progressors and healthy subjects. A. Heat map showing the relative abundance of the top ASVs significantly different between IgA+ and IgA- samples of CD progressors and healthy controls (ASVs=51, selected based on p-value) at age 2.5 B. at age 5. Each column represents an individual participant and each row represents an ASV. Figur [file 40168_2022_1429_MOESM1_ESM.zip › Figure S3.jpg]

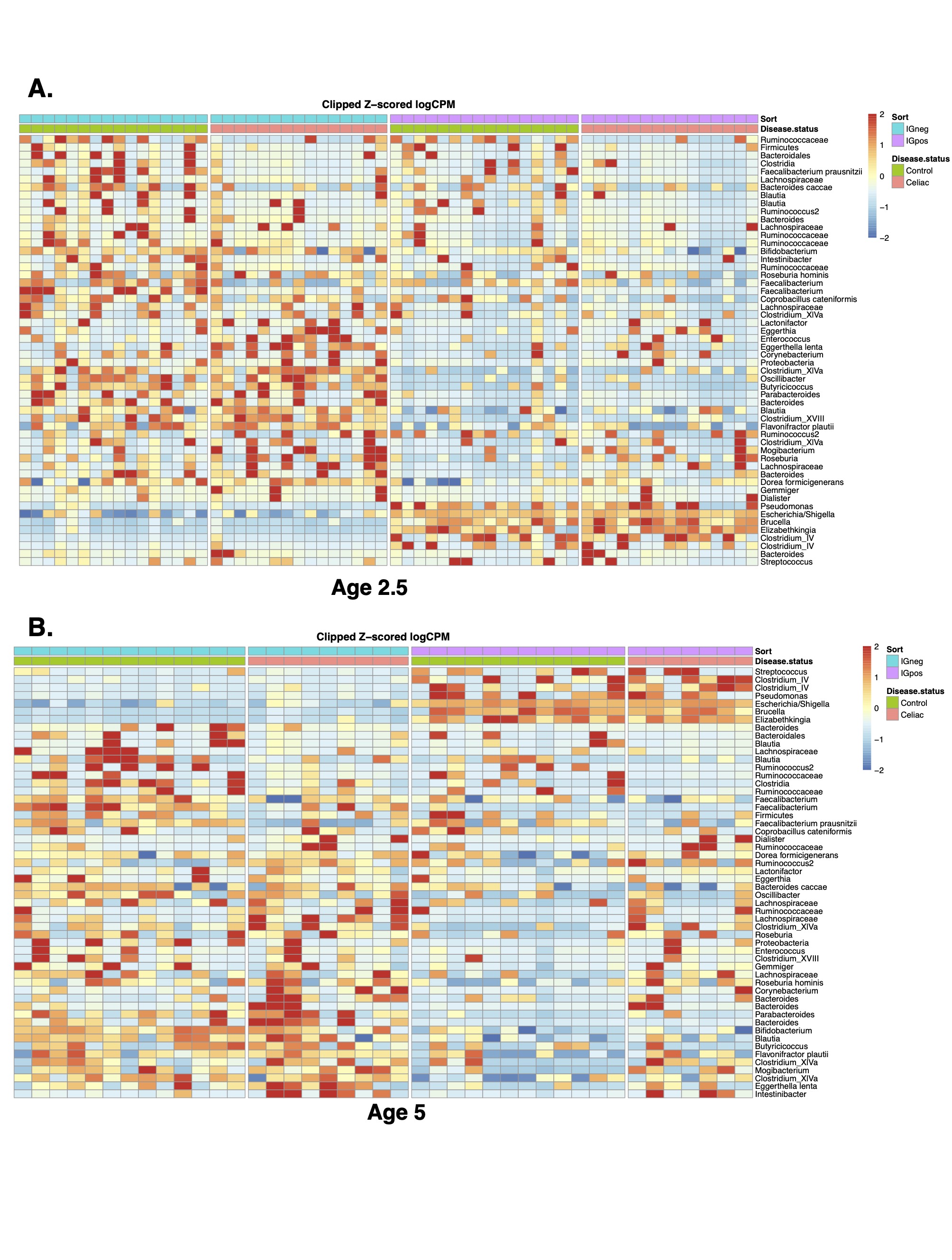

Supplement: Supplementary file 2 — Additional file 1: Figure S1. Flow chart describing CD progressors demographics and sample size. Figure S2. Violin plot representation of most enriched genus/species in CD progressors compared to healthy controls. A. Fold change in ASVs at age 2.5 CD progressors (left panel, n=15) and healthy controls (right panel, n=16). B. Fold change in ASVs at age 5 in CD progressors (left panel, n=10) and healthy controls (right panel, n=13). Figure S3. Gating strategy for IgA sequencing and analysis. A. Schematic overview of IgA-based fecal bacteria separation combined with 16S rRNA gene sequencing (IgA-seq) for stool samples from CD progressors and healthy controls. MACS: Magnetic-activated cell separation. B. Gating strategy for the isolation of IgA-/+ bacteria from the CD progressors and healthy controls’ fecal samples. C. Empirical Bayes quasi-likelihood F-tests analysis for comparing IgA-coated and non-coated gut microbiota ASVs in healthy controls (upper row) and CD progressors (lower row) at ages 2.5, and 5. Frequency: number of ASVs. FDR: False Discovery Rate. D. Empirical Bayes quasi-likelihood F-tests analysis for the comparisons of IgA-coated or non-coated gut microbiota ASVs between CD progressors and healthy controls (upper row: age 2.5 years old; lower row: age 5 years old). F. Box plots showing representative ASVs in which abundances were similar in the gut microbiota (presorting samples) but differently targeted by IgA at age 2.5. E. Violin plots showing representative ASVs in which abundances were similar in the gut microbiota (presorting samples) but differently targeted by IgA at age 5. Figure S4. Heat Map showing IgA target in CD progressors and healthy subjects. A. Heat map showing the relative abundance of the top ASVs significantly different between IgA+ and IgA- samples of CD progressors and healthy controls (ASVs=51, selected based on p-value) at age 2.5 B. at age 5. Each column represents an individual participant and each row represents an ASV. Figur [file 40168_2022_1429_MOESM1_ESM.zip › Figure S4.jpg]

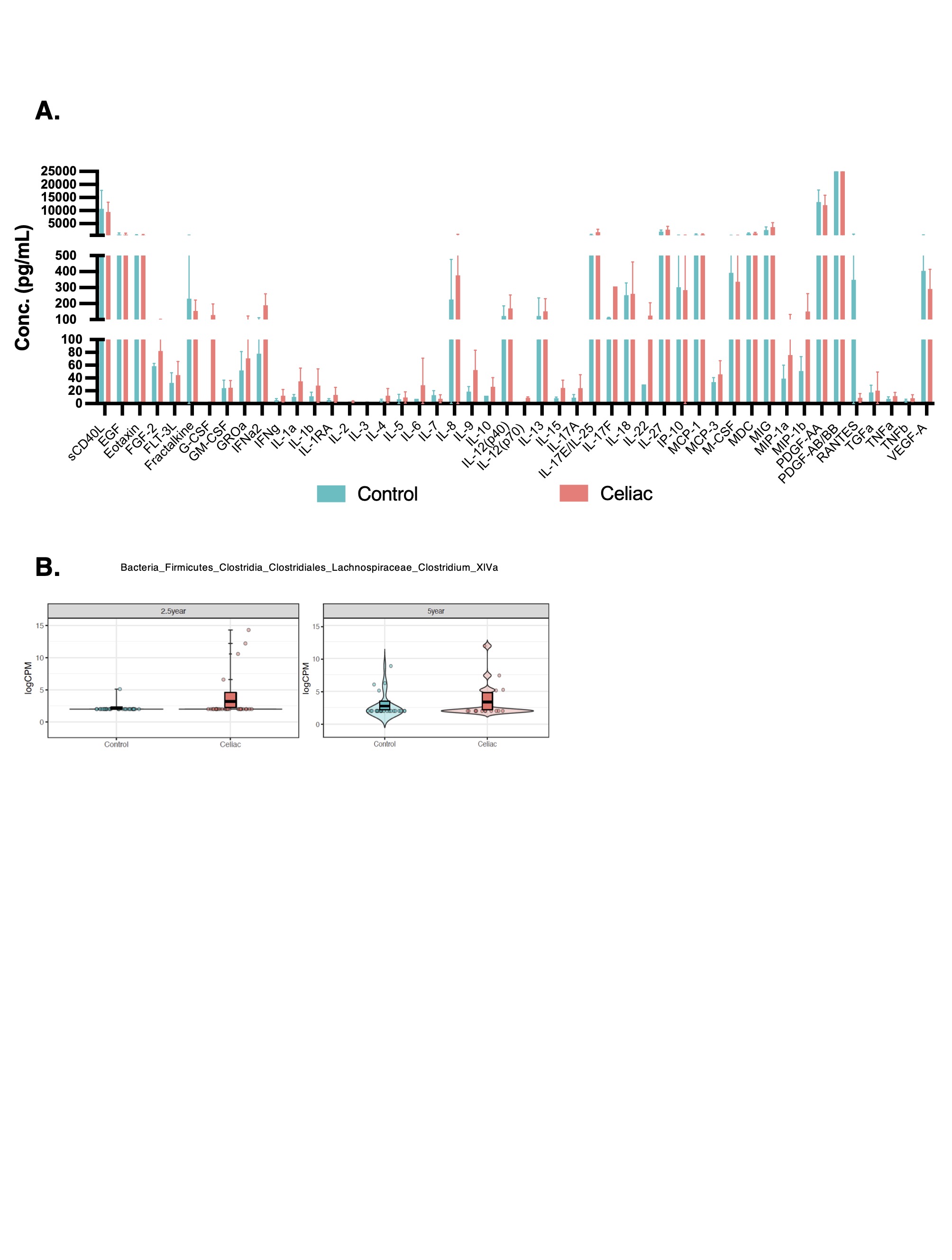

Supplement: Supplementary file 2 — Additional file 1: Figure S1. Flow chart describing CD progressors demographics and sample size. Figure S2. Violin plot representation of most enriched genus/species in CD progressors compared to healthy controls. A. Fold change in ASVs at age 2.5 CD progressors (left panel, n=15) and healthy controls (right panel, n=16). B. Fold change in ASVs at age 5 in CD progressors (left panel, n=10) and healthy controls (right panel, n=13). Figure S3. Gating strategy for IgA sequencing and analysis. A. Schematic overview of IgA-based fecal bacteria separation combined with 16S rRNA gene sequencing (IgA-seq) for stool samples from CD progressors and healthy controls. MACS: Magnetic-activated cell separation. B. Gating strategy for the isolation of IgA-/+ bacteria from the CD progressors and healthy controls’ fecal samples. C. Empirical Bayes quasi-likelihood F-tests analysis for comparing IgA-coated and non-coated gut microbiota ASVs in healthy controls (upper row) and CD progressors (lower row) at ages 2.5, and 5. Frequency: number of ASVs. FDR: False Discovery Rate. D. Empirical Bayes quasi-likelihood F-tests analysis for the comparisons of IgA-coated or non-coated gut microbiota ASVs between CD progressors and healthy controls (upper row: age 2.5 years old; lower row: age 5 years old). F. Box plots showing representative ASVs in which abundances were similar in the gut microbiota (presorting samples) but differently targeted by IgA at age 2.5. E. Violin plots showing representative ASVs in which abundances were similar in the gut microbiota (presorting samples) but differently targeted by IgA at age 5. Figure S4. Heat Map showing IgA target in CD progressors and healthy subjects. A. Heat map showing the relative abundance of the top ASVs significantly different between IgA+ and IgA- samples of CD progressors and healthy controls (ASVs=51, selected based on p-value) at age 2.5 B. at age 5. Each column represents an individual participant and each row represents an ASV. Figur [file 40168_2022_1429_MOESM1_ESM.zip › Figure S5.jpg]

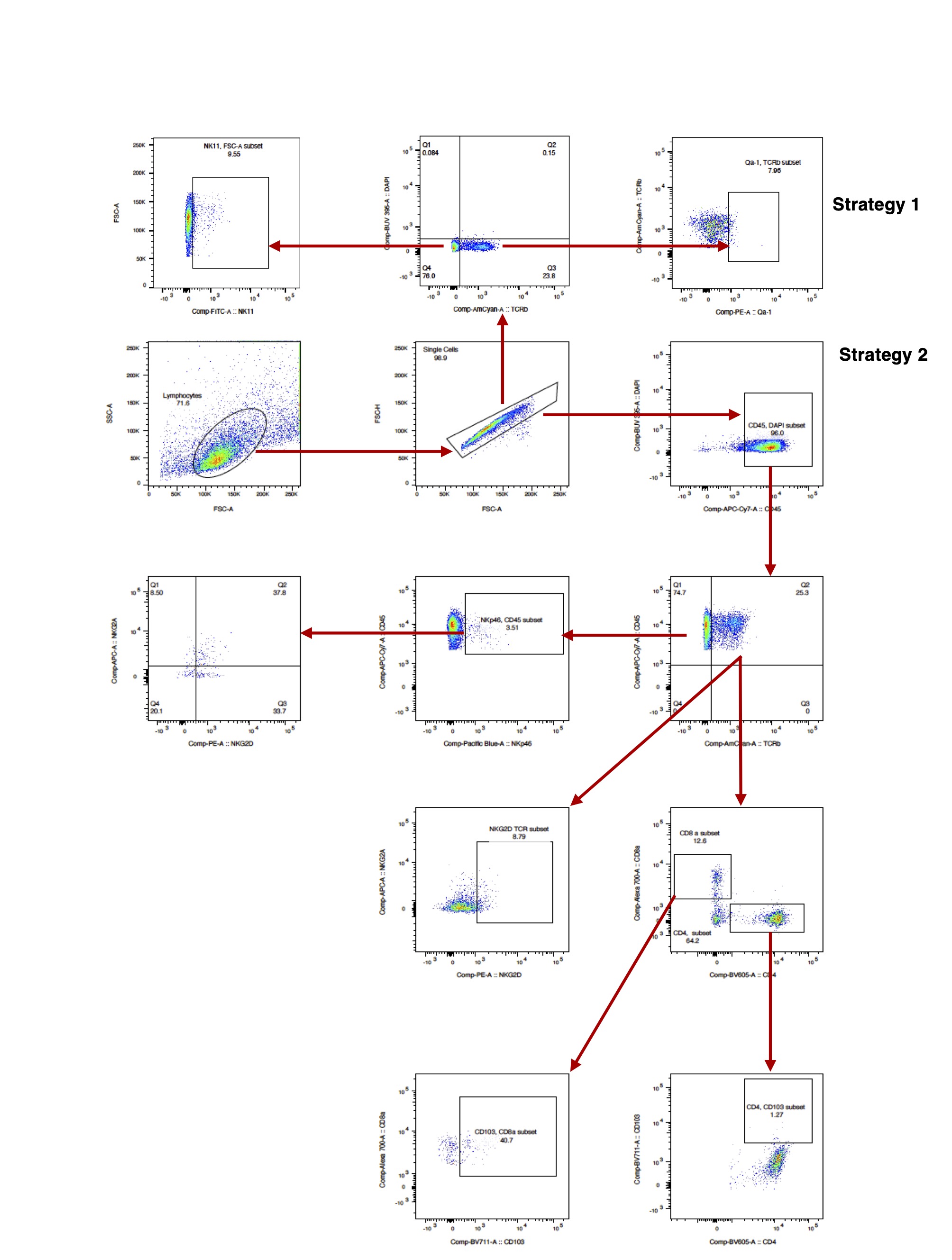

Supplement: Supplementary file 2 — Additional file 1: Figure S1. Flow chart describing CD progressors demographics and sample size. Figure S2. Violin plot representation of most enriched genus/species in CD progressors compared to healthy controls. A. Fold change in ASVs at age 2.5 CD progressors (left panel, n=15) and healthy controls (right panel, n=16). B. Fold change in ASVs at age 5 in CD progressors (left panel, n=10) and healthy controls (right panel, n=13). Figure S3. Gating strategy for IgA sequencing and analysis. A. Schematic overview of IgA-based fecal bacteria separation combined with 16S rRNA gene sequencing (IgA-seq) for stool samples from CD progressors and healthy controls. MACS: Magnetic-activated cell separation. B. Gating strategy for the isolation of IgA-/+ bacteria from the CD progressors and healthy controls’ fecal samples. C. Empirical Bayes quasi-likelihood F-tests analysis for comparing IgA-coated and non-coated gut microbiota ASVs in healthy controls (upper row) and CD progressors (lower row) at ages 2.5, and 5. Frequency: number of ASVs. FDR: False Discovery Rate. D. Empirical Bayes quasi-likelihood F-tests analysis for the comparisons of IgA-coated or non-coated gut microbiota ASVs between CD progressors and healthy controls (upper row: age 2.5 years old; lower row: age 5 years old). F. Box plots showing representative ASVs in which abundances were similar in the gut microbiota (presorting samples) but differently targeted by IgA at age 2.5. E. Violin plots showing representative ASVs in which abundances were similar in the gut microbiota (presorting samples) but differently targeted by IgA at age 5. Figure S4. Heat Map showing IgA target in CD progressors and healthy subjects. A. Heat map showing the relative abundance of the top ASVs significantly different between IgA+ and IgA- samples of CD progressors and healthy controls (ASVs=51, selected based on p-value) at age 2.5 B. at age 5. Each column represents an individual participant and each row represents an ASV. Figur [file 40168_2022_1429_MOESM1_ESM.zip › Figure S6.jpg]

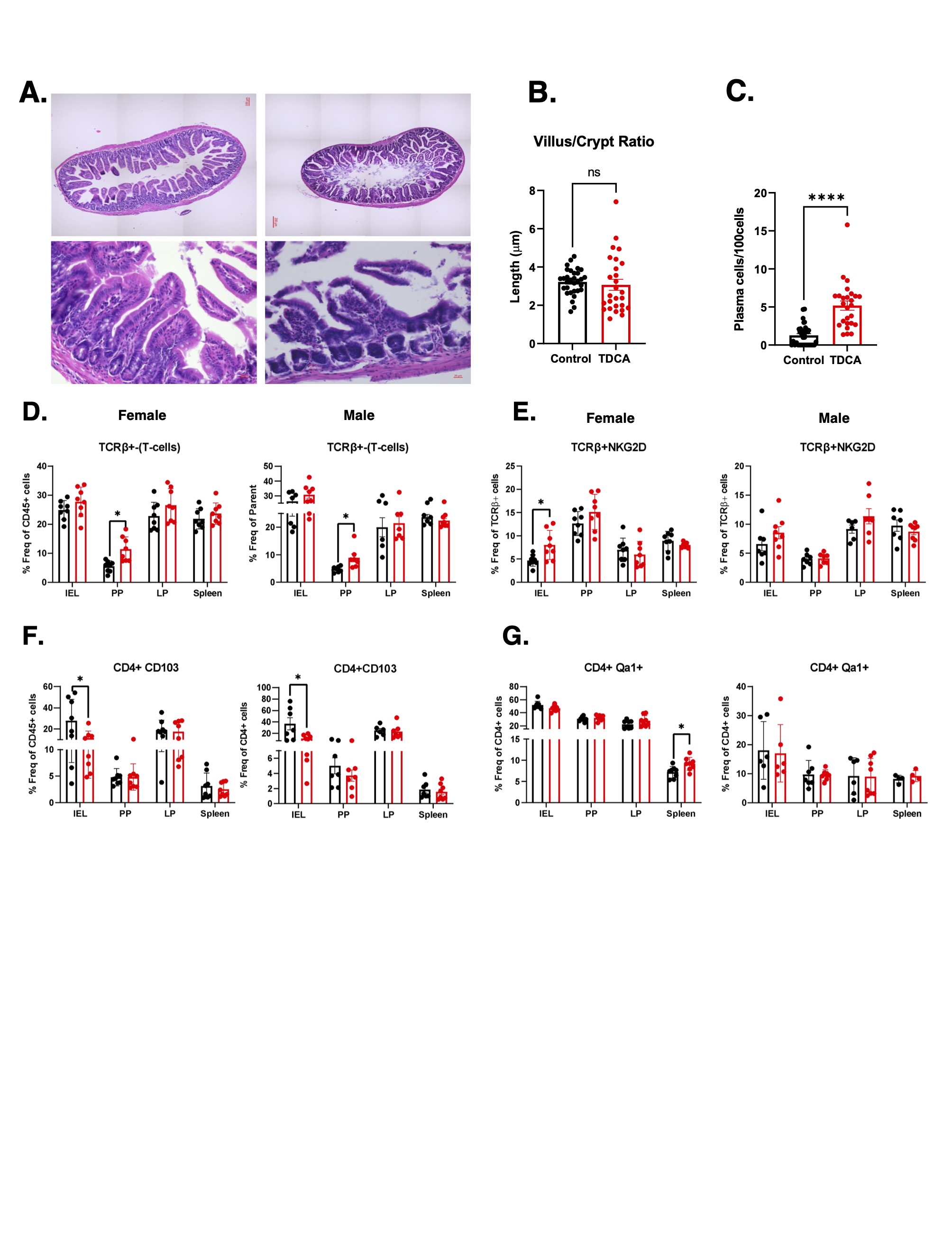

Supplement: Supplementary file 2 — Additional file 1: Figure S1. Flow chart describing CD progressors demographics and sample size. Figure S2. Violin plot representation of most enriched genus/species in CD progressors compared to healthy controls. A. Fold change in ASVs at age 2.5 CD progressors (left panel, n=15) and healthy controls (right panel, n=16). B. Fold change in ASVs at age 5 in CD progressors (left panel, n=10) and healthy controls (right panel, n=13). Figure S3. Gating strategy for IgA sequencing and analysis. A. Schematic overview of IgA-based fecal bacteria separation combined with 16S rRNA gene sequencing (IgA-seq) for stool samples from CD progressors and healthy controls. MACS: Magnetic-activated cell separation. B. Gating strategy for the isolation of IgA-/+ bacteria from the CD progressors and healthy controls’ fecal samples. C. Empirical Bayes quasi-likelihood F-tests analysis for comparing IgA-coated and non-coated gut microbiota ASVs in healthy controls (upper row) and CD progressors (lower row) at ages 2.5, and 5. Frequency: number of ASVs. FDR: False Discovery Rate. D. Empirical Bayes quasi-likelihood F-tests analysis for the comparisons of IgA-coated or non-coated gut microbiota ASVs between CD progressors and healthy controls (upper row: age 2.5 years old; lower row: age 5 years old). F. Box plots showing representative ASVs in which abundances were similar in the gut microbiota (presorting samples) but differently targeted by IgA at age 2.5. E. Violin plots showing representative ASVs in which abundances were similar in the gut microbiota (presorting samples) but differently targeted by IgA at age 5. Figure S4. Heat Map showing IgA target in CD progressors and healthy subjects. A. Heat map showing the relative abundance of the top ASVs significantly different between IgA+ and IgA- samples of CD progressors and healthy controls (ASVs=51, selected based on p-value) at age 2.5 B. at age 5. Each column represents an individual participant and each row represents an ASV. Figur [file 40168_2022_1429_MOESM1_ESM.zip › Figure S7.jpg]
